# Performance of diagnostic and predictive host blood transcriptomic signatures for Tuberculosis disease: A systematic review and meta-analysis

**DOI:** 10.1371/journal.pone.0237574

**Published:** 2020-08-21

**Authors:** Humphrey Mulenga, Chambrez-Zita Zauchenberger, Erick W. Bunyasi, Stanley Kimbung Mbandi, Simon C. Mendelsohn, Benjamin Kagina, Adam Penn-Nicholson, Thomas J. Scriba, Mark Hatherill

**Affiliations:** 1 South African Tuberculosis Vaccine Initiative (SATVI), Institute of Infectious Disease & Molecular Medicine and Division of Immunology, Department of Pathology, University of Cape Town, Cape Town, South Africa; 2 Vaccines for Africa Initiative (VACFA), School of Public Health & Family Medicine, University of Cape Town, Cape Town, South Africa; Food and Drug Administration, UNITED STATES

## Abstract

**Introduction:**

Host blood transcriptomic biomarkers have potential as rapid point-of-care triage, diagnostic, and predictive tests for Tuberculosis disease. We aimed to summarise the performance of host blood transcriptomic signatures for diagnosis of and prediction of progression to Tuberculosis disease; and compare their performance to the recommended World Health Organisation target product profile.

**Methods:**

A systematic review and meta-analysis of the performance of host blood mRNA signatures for diagnosing and predicting progression to Tuberculosis disease in HIV-negative adults and adolescents, in studies with an independent validation cohort. Medline, Scopus, Web of Science, and EBSCO libraries were searched for articles published between January 2005 and May 2019, complemented by a search of bibliographies. Study selection, data extraction and quality assessment were done independently by two reviewers. Meta-analysis was performed for signatures that were validated in ≥3 comparable cohorts, using a bivariate random effects model.

**Results:**

Twenty studies evaluating 25 signatures for diagnosis of or prediction of progression to TB disease in a total of 68 cohorts were included. Eighteen studies evaluated 24 signatures for TB diagnosis and 17 signatures met at least one TPP minimum performance criterion. Three diagnostic signatures were validated in clinically relevant cohorts to differentiate TB from other diseases, with pooled sensitivity 84%, 87% and 90% and pooled specificity 79%, 88% and 74%, respectively. Four studies evaluated signatures for progression to TB disease and performance of one signature, assessed within six months of TB diagnosis, met the minimal TPP for a predictive test for progression to TB disease.

**Conclusion:**

Host blood mRNA signatures hold promise as triage tests for TB. Further optimisation is needed if mRNA signatures are to be used as standalone diagnostic or predictive tests for therapeutic decision-making.

## Introduction

The World Health Organisation (WHO) has targeted 2035 to end tuberculosis (TB) and aims for 90% reduction in new TB cases and 95% reduction in TB deaths compared to 2015 levels [1]. Non-sputum triage, diagnostic, and predictive tests for TB may play a role in advancing TB control efforts. The WHO, in conjunction with the Foundation for Innovative New Diagnostics and the New Diagnostics Working Group of the Stop TB Partnership, has published Target Product Profiles (TPPs) for non-sputum biomarker triage, diagnostic, and predictive tests for progression from latent TB infection (LTBI) to TB disease [2–4]. The TPPs require minimum 90% sensitivity and 70% specificity for a triage test; 65% sensitivity and 98% specificity for a diagnostic test [2], and 75% sensitivity and 75% specificity for a test to predict progression from LTBI to active TB disease within two years [3, 4]. A new predictive test should also achieve a positive predictive value (PPV) of 5.8% given a 2% pre-test probability [3].

Current commercially available TB diagnostic tests are not optimal. *Mycobacterium tuberculosis* (MTB) culture, considered the gold standard, requires days to weeks to obtain a result from a reference laboratory, and is thus not ideal for rapid patient management [5, 6].Sputum smear microscopy has low sensitivity [7] ranging from 32% to 89% [8], resulting in a considerable proportion of active pulmonary TB patients being missed [9]. Sputum Xpert MTB/RIF and Xpert MTB/RIF Ultra have considerably better diagnostic performance [10, 11] than smear microscopy, but are similarly dependant on obtaining an adequate sputum sample; and need specialised laboratory equipment and a reliable power supply, which impedes routine screening in TB-endemic resource-limited settings [12].

Individuals with LTBI, defined by a positive tuberculin skin test (TST) or interferon-gamma release assay (IGRA), have a higher risk of progression to TB disease than MTB-uninfected people [13, 14]. However, only about 10–15% of people who test IGRA or TST positive will go on to develop TB disease [15, 16]. Predictive specificity of IGRA and TST for incident TB disease is poor (49.3% and 45% respectively) [14, 17]. While prevention of TB disease arising from LTBI is key to achieving WHO elimination targets [18], mass preventive therapy based on IGRA or TST screening in TB-endemic countries would need to treat a significant proportion of the population [19], most of them unnecessarily, which would be unaffordable and potentially ineffective, because re-infection would likely occur before programmatic coverage was complete.

In recent years, host blood transcriptomic signatures have offered a promising alternative as tests for both diagnosis of and prediction of progression to TB disease. These signatures have also improved our understanding of inflammatory processes associated with progression [20, 21] in individuals with MTB infection [22] and those with TB disease [23]. Host blood transcriptomic signatures have been shown to discriminate prevalent TB disease cases from MTB-uninfected and latently MTB-infected individuals, and individuals with other respiratory ailments [24]; and predict progression to TB disease in individuals with LTBI [25, 26].

Two systematic reviews have evaluated biomarkers for diagnosis of TB disease in children [27] and all age groups [28]. These systematic reviews did not include biomarkers for progression to TB disease; and did not include a meta-analysis, owing to heterogenous study designs, patient selection, and biomarker composition. Two additional studies performed re-analysis of patient-level data but did not perform a systematic review of signature performance as reported by the original studies. Warsinske et al [29] compared 16 signatures for TB diagnosis by recreating the original model of each signature and evaluated each signature across the datasets they had identified, and Gupta et al [30] conducted a meta-analysis of patient-level pooled data for signatures of incipient TB. We present a systematic review and meta-analysis of transcriptomic signatures which have been evaluated in independent validation cohorts. We aimed to summarise the performance of host blood transcriptomic signatures for diagnosis of and prediction of progression to TB disease in HIV-negative adults and adolescents; and to compare individual signature performance to the WHO TPP. This review is registered with the International Prospective Register of Systematic Reviews (PROSPERO), registration number CRD42017073817.

## Materials and methods

We conducted a systematic review according to standard guidelines [31, 32] (S1 Table). and designed the protocol prior to conducting the review [33] (http://dx.doi.org/10.1136/bmjopen-2018-026612).

### Study inclusion/exclusion criteria

We included studies evaluating host blood mRNA signatures for diagnosis of or prediction of progression to TB disease in HIV-negative adults and/or adolescents (≥ 12 years old) and published in English. Studies were restricted to those published between January 2005 and May 2019 to concentrate on recent evidence because of the fast pace at which the field of transcriptomics is changing and advancing. Only studies comparing TB disease cases versus MTB-uninfected controls, individuals with other diseases (ODs), or with LTBI; and using a microbiological reference standard of either sputum MTB culture, Xpert MTB/RIF, or smear microscopy for TB disease diagnosis, were eligible for inclusion. Studies of prediction of incident TB disease were required to be prospective, with a follow-up period of at least six months; and enrolling either TB contacts, latently MTB-infected, or uninfected individuals. Studies were excluded if: conducted in animals; were in children younger than 12 years, did not report sensitivity and specificity, did not allow recreation of a 2 x 2 contingency table for calculation of test performance, and where authors did not respond to enquiries for data within four weeks of inquiry. Unpublished reports and conference proceedings were excluded due to absence of peer review and difficulty in obtaining data.

### Literature search

Medline via PubMed, Scopus, Web of Science, and EBSCO databases were searched for relevant studies. The search strategy developed on PubMed was adapted to other databases and was as follows:

((((((Tuberculosis [MeSH] OR Mycobacterium tuberculosis [MeSH] OR (Tuberculosis OR TB OR Mycobacterium tuberculosis OR MTB))) AND ((Diagnosis [MeSH] OR Diagnosis [subheading] OR Prognosis [MeSH] OR (Diagnosis OR diagnostic OR detect* OR predict* OR prognosis OR prognostic OR screen*)))) AND ((Biomarkers/Blood [MeSH] OR RNA/Blood [MeSH] OR Transcription, Genetic [MeSH] /etiology/genetics/immunology OR (Blood Biomarker OR blood biomarkers OR bio-signature OR gene expression OR genetic transcription OR host blood OR immune marker OR immunologic marker OR Ribonucleic Acid OR RNA OR signature OR surrogate endpoint OR surrogate marker OR transcriptome OR transcriptomic)))) AND ((Area under Curve [MeSH] OR Sensitivity and Specificity [MeSH] OR (Area under curve OR area under curves OR AUC OR receiver operating characteristic OR ROC OR Accuracy OR Performance OR sensitivity OR specificity)))) AND (Humans[Mesh])) AND (("2005/01/01"[Date—Publication]: "2019/05/31"[Date—Publication])). Additionally, bibliographies of included papers were scrutinised for potential papers that were missed by the search terms.

### Study selection

**Fig 1:**
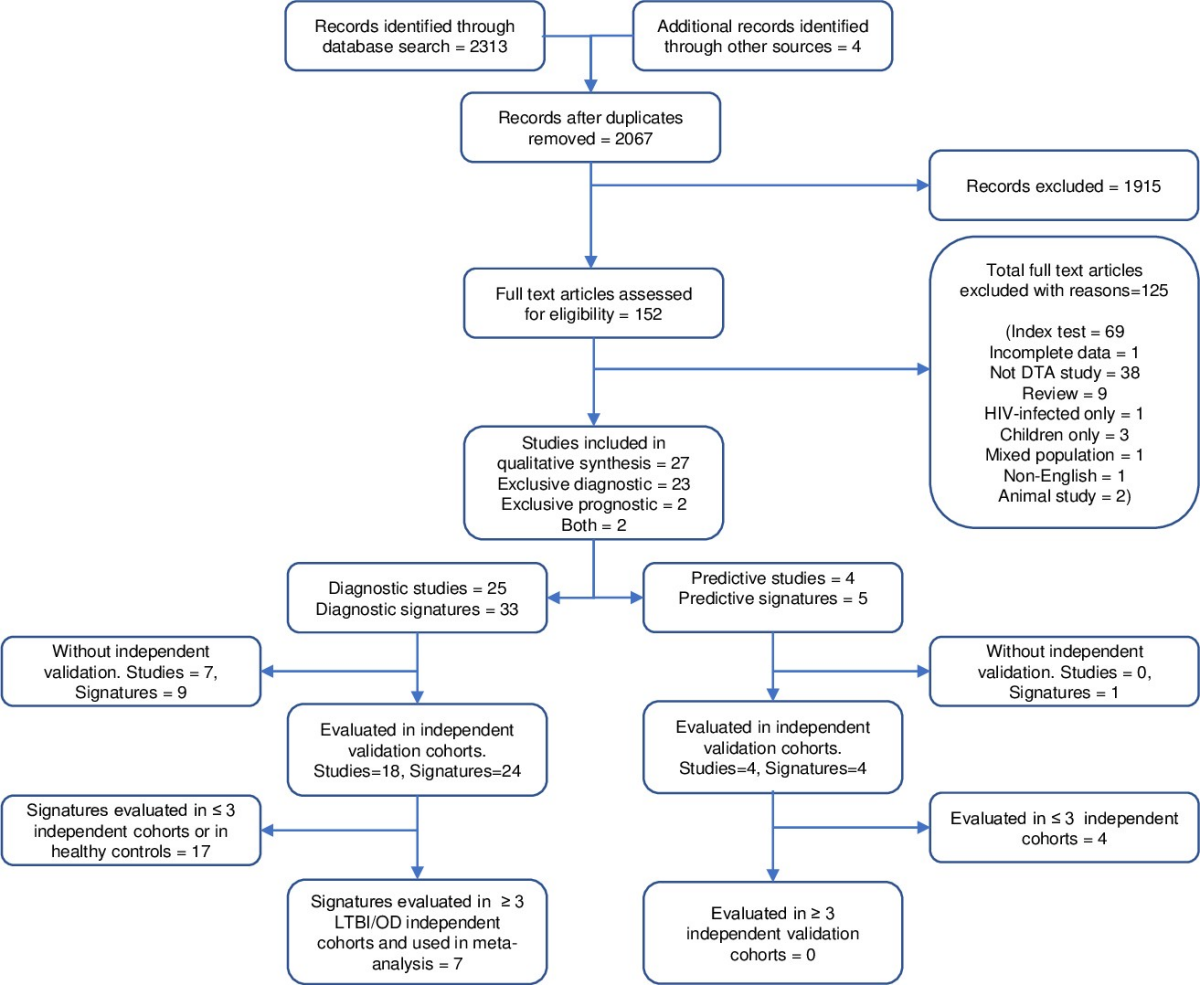
Flow of studies in the review of transcriptomic signatures for diagnosing and predicting progression to TB disease. DTA; diagnostic test accuracy, LTBI; Latent TB infection, OD; Other diseases.

Two reviewers (HM and CZZ) independently screened search outputs for eligible studies. Publications were first screened by title and abstract, and thereafter by full text. Articles were independently categorised as either (i) selected, (ii) not selected, or (iii) pending. The two reviewers conferred to resolve any disagreements about pending publications, and if a consensus could not be reached, discrepancies were adjudicated by a third reviewer (BK). Fig 1 depicts the study selection process.

### Data extraction and management

Data metrics were extracted separately from the relevant articles and double-entered into a Microsoft SQL Server 2012 database to facilitate electronic comparison of data between reviewers. For each biomarker test, we extracted the reported performance data; sensitivity, specificity, true positives (TP) false positives (FP), true negatives (TN), and false negatives (FN); as well as details of the study design, population, and index and reference test characteristics using a customised data extraction form (S1 File). We requested missing data from authors by email.

### Appraisal of methodological quality of studies

HM and CZZ independently assessed the quality of included studies using a customised form based on the Quality Assessment of Diagnostic Accuracy Studies-2 (QUADAS-2) [34] assessment tool (S2 File, Methods in S3 File). BMK provided adjudication where a disagreement occurred. For each of the four domains in QUADAS-2, namely “patient selection”, “index test”, “reference standard” and “flow and timing”; risk of bias was scored as “low” if all responses in that domain were answered as “yes”, “high” if any of the responses were answered as “no” or “unclear” and “unclear” if it was unclear for all the responses. We judged “low applicability concerns” for the patient selection domain if a clinically relevant control population was used; OD for diagnostic studies and LTBI or household TB contacts for predictive studies and “high applicability concerns” if other populations were used. For other domains, we judged “low applicability concerns” if all signalling questions in that domain were answered as “yes”, “high applicability concerns” if any question was answered as “no” and “unclear applicability concerns” if answered as such.

### GRADE quality of evidence

We used the “Grading of Recommendations Assessment, Development and Evaluation” (GRADE) approach to judge the quality of evidence. Classification of the quality of evidence was based on study design in conjunction with the five factors that affect study quality; study limitations, indirectness, inconsistency, imprecision, and publication bias [35].

### Data synthesis and analysis

We extracted data to construct 2 x 2 contingency tables of reference test versus index test results. TB positive and TB negative were defined as participants with and without TB disease respectively, based on the reference standard. Forest plots of sensitivity and specificity with 95% confidence intervals for each signature were created using RevMan 5.3 [36]. Each entry in the forest plot represents a signature that was evaluated in a distinct cohort. Several signatures were tested in multiple cohorts and reported in multiple studies. In naming individual studies, we used the first author name, year of manuscript publication, and a sequential letter representing a specific cohort. Similarly, the signature naming convention was first author name, number of genes, and year of publication.

We reported the index test results as TP, FP, TN and FN. If not explicitly reported, TP, FP, TN, and FN were estimated from the reported sensitivity and specificity and total number of TB positive individuals and controls. Similarly, sensitivity and specificity were reported, or calculated if the data were available or obtained from the authors. We also calculated the PPV and negative predictive values (NPV) at 2% pre-test probability for predictive signatures.

Signatures used for both diagnosis and prediction of TB disease are presented in separate forest plots. Each evaluation of a signature in a different population, or of different signatures in the same population, is shown as a separate entry (or entries) in the forest plot. If the same signature was reported using different models, the best performing model was included in the analysis. Similarly, if a study reported several signatures with the same number of genes in the same population, only the best performing signature was chosen.

Considerable clinical and methodological heterogeneity was anticipated due to reporting of multiple signatures in different populations. Therefore, we did not perform meta-analysis on all signatures in all studies. In order to address this heterogeneity and compare relative performance of signatures, we performed meta-analysis only for signatures that were evaluated in at least three comparable cohorts with the same control population. Meta-analysis was conducted in STATA 11, using hierarchical logistic regression. We used the bivariate random effects model to calculate summary sensitivity and specificity with the corresponding 95% CI [37], and to create summary receiver operating characteristic curves for each signature. Heterogeneity in the diagnostic or predictive performance of the signatures was assessed by visual inspection of the forest plots and the I^2^ statistic.

## Results

### Search results

Our search term returned 2,313 reports of which 27 [23–26, 38–62] satisfied all the pre-specified inclusion criteria (Fig 1). Twenty-three of the 27 studies reported exclusively on diagnostic performance of mRNA signatures for discriminating TB disease cases from controls with or without ODs, and controls with or without LTBI. Two studies reported exclusively on predictive performance for progression to TB disease [26, 55]; and two studies reported both diagnostic and predictive performance [25, 60]. A total of 35 transcriptomic signatures incorporating 1,027 genes were identified (S2 Table). Forty-two of the 1,027 genes were employed in at least three or more transcriptomic signatures and Fc gamma receptor 1A (FCGR1A) was the most frequently utilised gene (Fig 1 in S3 File)

### Quality of diagnostic studies

**Fig 2:**
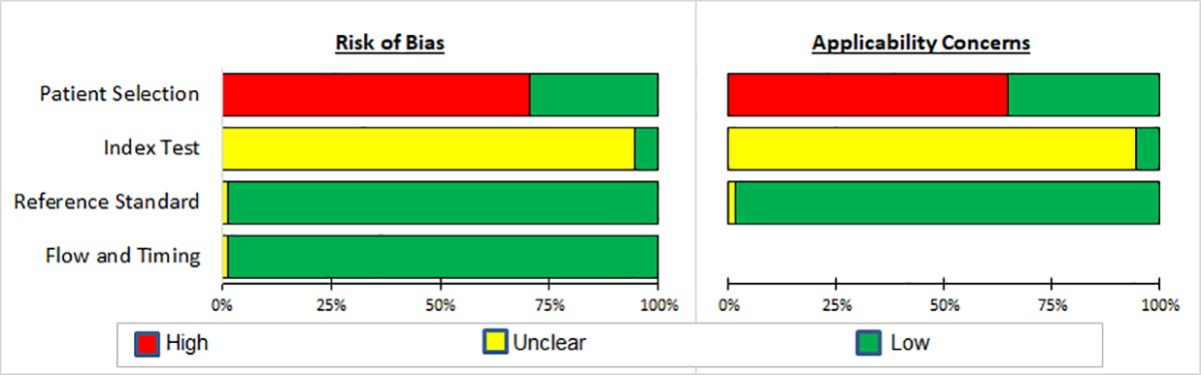
Summary of results of QUADAS-2 assessment of diagnostic studies in clinically relevant independent validation cohorts with other diseases.

**Fig 3:**
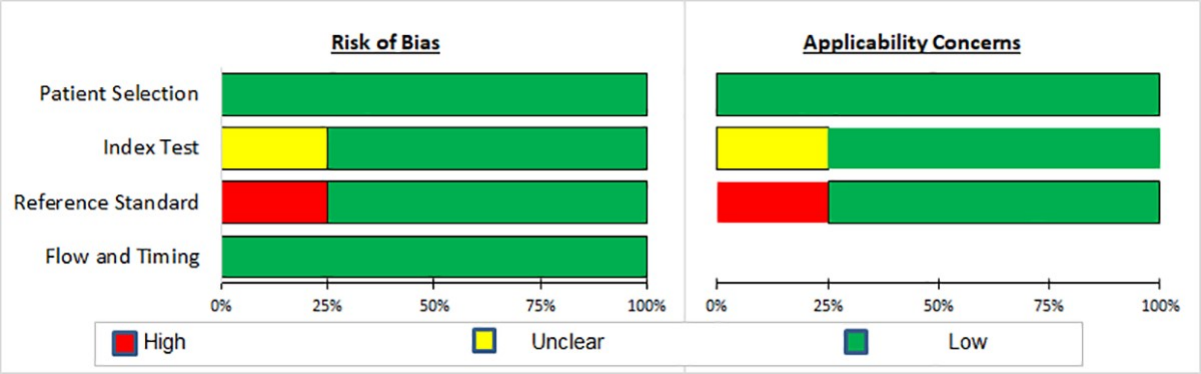
Summary of results of QUADAS-2 assessment in studies of prediction to TB disease in independent validation cohorts.

Four studies [42, 44, 58, 60] employed a cohort design, four [23, 38, 51, 52] a cross sectional design, and the remainder employed a case-control design. All studies had a reference standard of either smear microscopy [50], Xpert/MTB RIF [41] or MTB culture; Table 1 in S3 File. One study [46] did not specify the type of reference test used. Eight [23–25, 38, 44, 45, 49, 56, 59, 60] of the 18 studies evaluated signatures in populations with ODs. Only one study [43] made reference to blinding of the index test readers. Bias in patient selection arose in most studies due to case-control design and non-reporting of sampling method, resulting in a non-representative spectrum of patients. Bias in the index test resulted from the lack of reported blinding in the index test interpretation. Bias in the reference standard was minimal since 97% (68/70) of the entries used MTB culture as a reference standard which can be considered objective (Fig 2; Figs 2 and 3 in S3 File).

### Quality of studies for prediction of progression to TB disease

**Fig 4:**
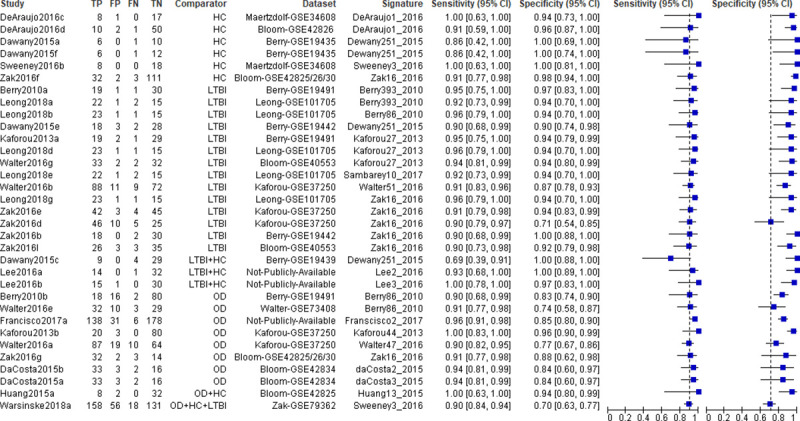
Forest plots of sensitivity and specificity of transcriptomic signatures for diagnosis of TB disease that met at least one minimum TPP performance criterion in independent validation cohorts. HC; Healthy controls, LTBI; Latent TB infection, OD; Other diseases. Vertical dashed lines correspond to 90% sensitivity and 70% specificity.

All studies [25, 26, 55, 60] validated signatures in participants from prospective independent cohorts, although two studies [25, 26] were case-control studies nested in prospective cohorts (Table 2 in S3 File). Participants from three studies did not all receive the same reference standard [26, 55, 60] and blinding was only stated in two studies [25, 26] (Fig 3; Fig 4 in S3 File).

### Performance of mRNA signatures for TB Diagnosis

**Fig 5:**
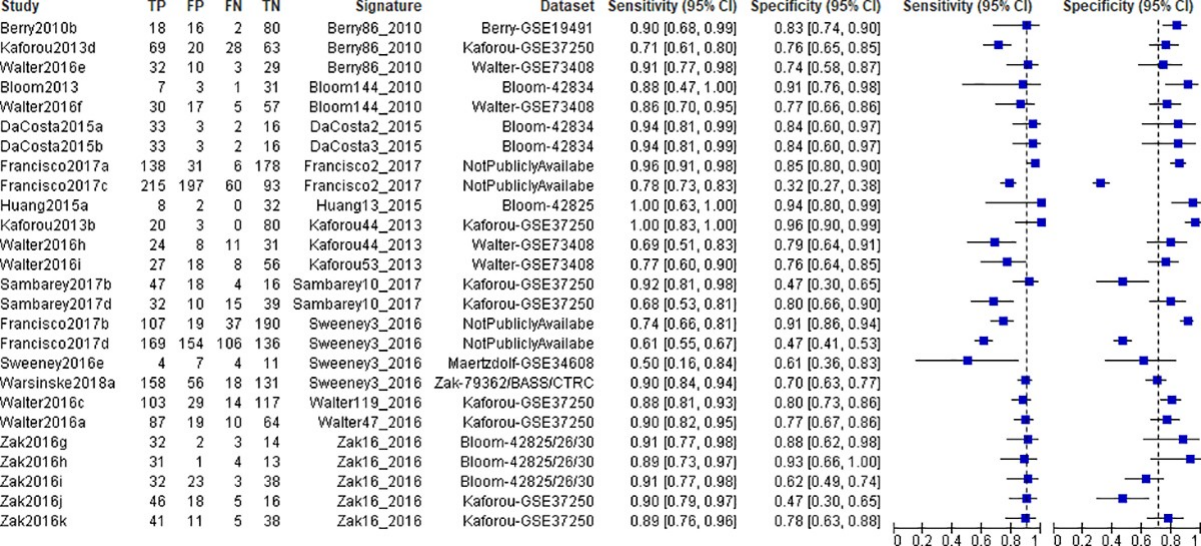
Forest plots of sensitivity and specificity of transcriptomic signatures for diagnosis of TB disease in independent validation cohorts of clinically relevant populations with other diseases. Vertical dashed lines correspond to 90% sensitivity and 70% specificity.

**Fig 6:**
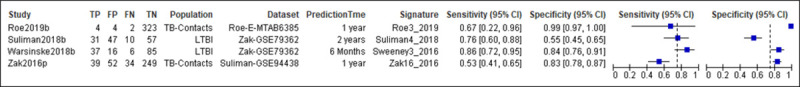
Forest plots of sensitivity and specificity of transcriptomic signatures for prediction of progression to TB disease in independent validation cohorts. Vertical dashed lines correspond to 75% sensitivity and 75% specificity. Prediction time is the time to TB disease used in each study.

Eighteen studies evaluated 24 transcriptomic signatures for diagnosis of TB disease in 70 different independent validation cohorts (Fig 5 in S3 File). Individual signatures displayed substantial variation in diagnostic performance with sensitivity ranging from 50%-100% and specificity ranging from 32%-100%. The observed heterogeneity (I^2^ = 0.99) in study design and signature performance precluded pooling of diagnostic accuracy estimates for all signatures. Nine signatures were not evaluated in independent validation cohorts and hence excluded (Fig 6 in S3 File).

Thirty-three entries (46.5%) representing 17 different signatures in 12 studies met at least one TPP minimum performance criterion in independent validation sets containing uninfected controls, LTBI, ODs, or a combination of these populations (Fig 4). Signature performance ranged between 69%-100% for sensitivity, and between 70%-100% for specificity.

Ten studies evaluated 12 signatures for diagnosis of TB disease in clinically relevant populations with ODs (Fig 5). Signature performance ranged between 50%-100% for sensitivity, and between 47%-96% for specificity. Seven of these signatures; Berry86_2010, daCosta2_2015, daCosta3_2015, Francisco2_2017, Kaforou44_2015, Walter47_2016, and Zak16_2016 met the WHO-recommended minimal TPP for a triage test. None of these signatures met the WHO minimal TPP for a diagnostic test in the OD population.

### Meta-analysis of performance of mRNA signatures for diagnosis of TB disease

**Fig 7:**
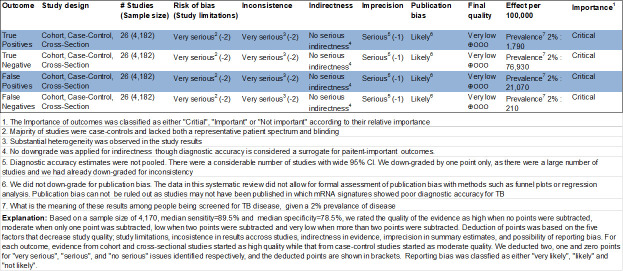
GRADE evidence profile: Transcriptomic signatures for the diagnosis of TB in clinically relevant populations with other diseases only.

Ten studies included seven signatures that were validated for diagnosis of TB in at least three comparable cohorts including either LTBI or OD populations. Signature diagnostic performance results are shown in Table 1. We excluded signatures validated exclusively in populations with uninfected controls, but included signatures evaluated in LTBI or OD populations that also included uninfected controls. Four signatures were validated in exclusively LTBI populations and six signatures were validated exclusively in OD populations; and two signatures in both LTBI and OD populations. Among the six signatures validated in LTBI cohorts, three signatures with similar diagnostic accuracy (Berry393_2010, Kaforou27_2013, Zak16_2016) showed pooled sensitivity and specificity that met the minimal WHO TPP for a non-sputum biomarker triage test in this population. Summary receiver operating characteristic curves for the signatures in OD populations are shown as Figs 7a, 7b and 7c in S3 File. Heterogeneity in the signature performance was not explained by comparison group (I^2^ = 0.98 for all OD, I^2^ = 0.95 for all LTBI, I^2^ = 0.90 for all healthy controls,). Zak16_2016 which used the TPP bench-mark of 90% sensitivity had a lower I^2^ of 0.56 compared to Sweeny3_2016’s I^2^ of 0.93. Some of the heterogeneity was explained by whether the evaluation used the TPP benchmarks and by composition of the control population.

**Table 1:** Meta-analysis of performance of transcriptomic signatures for diagnosis of TB disease in independent validation cohorts that included LTBI or other disease populations.

Signature	Study Entry ID	TB/ Control	Control Group	Sensitivity % (95% CI)	Specificity % (95% CI)
Berry393_2010	Berry2010a	20/31	LTBI	90 (83, 94)	92 (82,96)
	Kaforou2013c	97/83	LTBI		
	Leong2018a	24/16	LTBI		
	Walter2016d	35/35	LTBI		
Dawany251_2015	Dawany2015b	29/38	LTBI	82 (70, 90)	95 (88, 98)
	Dawany2015c	13/29	LTBI*		
	Dawany2015d	21/33	LTBI*		
	Dawany2015e	20/31	LTBI		
Kaforou27_2013	Kaforou2013a	20/31	LTBI	95 (87, 98)	93 (85, 97)
	Leong2018d	24/16	LTBI		
	Walter2016g	35/35	LTBI		
Samabarey10_2017	Leong2018e	24/16	LTBI	83 (75, 88)	92 (85, 96)
	Sambarey2017a	51/36	LTBI		
	Sambarey2017c	47/47	LTBI		
Sweeney3_2016	Leong2018f	24/16	LTBI	89 (84, 92)	72 (66, 78)
	Warsinske2018a	176/187	LTBI+		
	Sweeney2016d	46/25	LTBI		
Zak16_2016	Leong2018g	24/16	LTBI	91 (86, 94)	90 (72, 97)
	Zak2016a	21/21	LTBI		
	Zak2016b	20/30	LTBI		
	Zak2016d	51/35	LTBI		
	Zak2016e	46/48	LTBI		
	Zak2016l	29/38	LTBI		
Berry86_2010	Berry2010b	20/96	OD	84 (68, 93)	79 (73, 84)
	Kaforou2013d	97/83	OD		
	Walter2016e	35/39	OD		
Sweeney3_2016	Francisco2017b	275/290	OD	74 (57, 86)	71 (49, 86)
	Francisco2017d	144/209	OD		
	Sweeney2016e	8/18	OD		
	Warsinske2018a	176/187	OD+		
Zak16_2016	Zak2016g	35/16	OD	90 (85, 93)	74 (56, 86)
	Zak2016h	35/14	OD		
	Zak2016i	35/61	OD		
	Zak2016j	51/34	OD		
	Zak2016k	46/49	OD		

LTBI; Latent TB infection, OD; Other diseases, HC; Health control

*Includes some HCs, +Consists of HCs, ODs and LTBIs

### Performance of mRNA signatures for prediction of progression to TB disease

Four studies evaluated five signatures for prediction of progression to TB disease in independent validation cohorts with LTBI and uninfected controls. Two of these studies evaluated LTBI populations only; one study evaluated TB contacts only; and one study evaluated both TB contacts and LTBI populations (Fig 6). The time window between signature measurement and TB diagnosis reported in these studies was not consistent and ranged from 6 months to 24 months before TB diagnosis. Since differential gene expression becomes more pronounced as individuals approach TB diagnosis [21, 25, 26], this variable precluded direct comparison of signature performance for prediction of progression to TB disease. In LTBI populations, signature performance ranged between 76%-86% for sensitivity; and between 55%-84% for specificity. Signatures evaluated in TB contacts showed between 53%-67% sensitivity and 83%-99% specificity. The Sweeney3_2016 signature met the TPP performance criterion (PPV ≥ 5.8% and 75% sensitivity and 75% specificity) for a test to predict progression when measured within 6 months of TB diagnosis. Performance of the other signatures was not reported for this 6-month predictive interval (time to TB disease) and none of the other signatures met the TPP performance criterion over longer predictive intervals. Regardless, two other signatures, assessed within 12 months of TB diagnosis, also achieved a PPV ≥ 5.8% (Table 3 in S3 File).

### GRADE evidence profile

We followed a previously published GRADE guideline [35] to assess the quality of the body of evidence and produced a GRADE evidence profile (Fig 7 and Fig 8 in S3 File). The overall quality of evidence supporting the estimates of sensitivity and specificity of mRNA signatures for the diagnosis of TB disease was rated as “very low”. Consequently, very low confidence is placed in the estimates obtained from pooling studies in meta-analysis. Similarly, the quality of evidence for studies of progression to TB disease was also very low.

## Discussion

The New Diagnostics Working Group Strategic Framework 2016–2020 aims to “achieve early and universal diagnosis of all patients with all forms of TB to foster progress towards TB elimination, by making appropriate and affordable diagnostic solutions available at the right setting” [63]. Comparison of signature performance in similar populations and under similar conditions is critical to down-select candidate biomarkers for development as rapid point-of-care (POC) tests. Future implementation decisions hinge on test performance in clinically relevant populations under field conditions, in addition to feasibility and cost considerations that contribute to the assessment of impact and public health value.

We show that of the 17 mRNA signatures that met at least one minimal TPP performance criterion, 11 were validated in populations including either uninfected controls, LTBI, or both, and four were validated exclusively in populations with ODs. Four signatures validated in the uninfected control category (Dewany251_2015, Sweeney3_2016, Zak16_2016, and Lee2_2016) have potential as diagnostic tests while the remainder show potential as triage tests. The signatures in the LTBI/uninfected control category that are yet to be validated in populations with ODs such as DeAraujo1_2016, Lee2_2016, Lee3_2016, and Sambarey10_2017 should also be validated in such populations to confirm robustness of diagnostic accuracy and allow comparison with the signatures above.

Although TB triage tests might be used in community mass screening campaigns or contact investigations that include uninfected individuals or asymptomatic individuals with LTBI; the greatest need is for TB diagnostic tests that discriminate TB from ODs among symptomatic individuals seeking health care. We also recognise that evaluation of novel biomarkers in cohorts of carefully selected TB cases and uninfected controls tends to over-estimate performance. In this regard, diagnostic evaluation of signatures in populations that exclusively included LTBI or uninfected controls would be considered inferior to validation of performance in clinically relevant cohorts that included individuals with other respiratory diseases. The OD category in several of these studies did not include symptomatic individuals presenting to healthcare facilities with suspected TB, but rather individuals with systemic lupus erythematosus, sarcoidosis, or other infrequently encountered conditions. None of the signatures validated in cohorts with ODs met the minimum WHO TPP for sensitivity and specificity of a diagnostic test. However, seven signatures; Berry86_2010, daCosta2_2015, daCosta3_2015, Francisco2_2017, Kaforou44_2015, Walter47_2016, and Zak16_2016 met the minimum WHO TPP for a triage test. The findings suggest that these seven signatures could be further evaluated as TB triage tests under field conditions; and that feasibility and unit cost-effectiveness as potential rapid POC tests would be a consideration for further clinical development. Other signatures that approached but did not meet the TPP may also warrant further validation in side-by-side comparison studies. Signatures with smaller number of genes which may be more adaptable to a POC device could be given preferential consideration for clinical validation and development. Our findings also suggest that further improvement in performance of existing signatures, or even further discovery of new signatures with improved performance, would be necessary for clinical development of a non-sputum TB diagnostic test. For instance, incorporating covariates such as age and sex in the models.

Validation of diagnostic tests in several geographically different populations is important to confirm robustness. Relatively few signatures were validated in at least three comparable cohorts of a similar population and were eligible for meta-analysis. Only one of the signatures (Zak16_2016), validated in multiple cohorts that included ODs such as pneumonia, lung cancer, sarcoidosis, or systemic lupus erythematosus, met the minimum WHO TPP performance criteria for a triage test in meta-analysis and none met the minimal TPP for a diagnostic test in the meta-analyses. The finding suggests that this signature has potential as a rapid POC test and should be considered for clinical development upon validation under field conditions. Similarly, other signatures approaching the minimum TPP target in meta-analysis should be considered for field validation.

Tests that will accurately predict which individuals with LTBI will develop TB disease are needed to ensure that preventive treatment can be targeted for those individuals at increased risk of incident TB disease, while saving those individuals at lowest risk from the cost, burden, and side-effects of unnecessary intervention. Current tests for MTB infection, including IGRA and TSTs, are poor predictors because of their low specificity for incident TB disease. It is not cost-effective to treat the estimated two billion individuals latently infected with MTB worldwide and therefore preventive therapy targeted with a more specific biomarker may be a more feasible alternative [64]. Predictive signatures might be used in community-level TB mass-screening campaigns or contact investigations, but might also be useful in symptomatic individuals who have been investigated and found not to have active TB disease at the time of testing. Only one of the four signatures (Sweeney3_2016) met the minimum TPP criteria for both PPV ≥ 5.8% and 75% sensitivity and 75% specificity for a 6-month period prior to TB diagnosis, although this was the only signature for which performance within six-months of TB diagnosis was reported. The other studies reported signature performance one or two years prior to TB diagnosis. It is thus not clear how these signatures would perform during the six-month period before TB diagnosis. These results highlight that more studies of predictive performance are necessary. It is also evident that a two-year predictive horizon may be overly optimistic for prediction of progression to TB disease as progression is very variable in occurrence.

One of the challenges of this systematic review and meta-analysis was that most studies did not specify whether the signature being tested was intended for triage, diagnostic, or predictive use, neither did they benchmark performance of signatures against the WHO TPPs. For example, if the goal is to discover and validate a TB triage test, a study should report specificity at 90% sensitivity or higher, to allow comparison with other novel biomarkers against this standard. Similarly, if the goal is to discover and validate a TB diagnostic test, the study should ideally report sensitivity at 98% specificity or higher; and studies aiming to discover and validate a test to predict progression to TB disease should report sensitivity at 75% specificity or higher, or PPV and NPV along with the prediction time horizon. However, it must be noted that the differences in the benchmarks are partially because some of the studies were completed before publication of the TPPs. Secondly, we found that many studies were sub-optimally designed and used control populations without clinical relevance. As observed in a previous systematic review [28], we found that several of transcriptomic signatures for TB diagnosis were discovered but not validated in independent representative cohorts. We also found that a number of diagnostic accuracy studies did not conform to the reporting guidelines for diagnostic test accuracy (DTA) studies stipulated in the “Standards for the Reporting of Diagnostic Accuracy Studies” (STARD) [65]. In several studies, cardinal data on study design, patient selection, numbers of participants in each group, and diagnostic performance data such as sensitivity and specificity with their corresponding confidence intervals (CIs) that would enable reproduction of the study were not reported. This is a major drawback in synthesising the body of evidence on DTA studies and thus compliance to STARD in designing DTA studies and reporting their findings cannot be over-emphasised.

### Strengths and limitations of the study

We used an inclusive time frame of January 2005 to May 2019 to include the period in which we believe all transcriptomic TB biomarker studies were published. We also developed a protocol prior to performing the systematic review that explicitly stated a rigorous search strategy and clear inclusion/exclusion criteria. Unlike previous systematic reviews, our review includes evaluation of signatures for predicting progression to TB disease and a meta-analysis.

Some signatures were designed to optimise sensitivity while others were designed to optimise specificity. This may have introduced bias in the pooled estimates of sensitivity and specificity in the meta-analysis, and difficult to compare signature performance. Restricting included studies to those conducted in HIV-negative adults and adolescents may have excluded signatures with superior diagnostic performance in studies conducted in children or in HIV-positive individuals. Additionally, language selection bias cannot be ruled out since we only included studies reported in English. We did not formally assess publication bias as current methods are not suitable for DTA studies [66].

Heterogeneity of study design and reliance on reported data makes it impossible to fairly compare signature performance. A major finding of this study and limitation is the very low quality of evidence: preponderance of case control studies, spectrum bias and narrow geography. This highlights the need for high quality, prospective studies, with relevant populations of symptomatic clinic attendees, mass screening endemic community population or high-risk populations such as household TB contacts which minimise spectrum bias, and from multiple geographies.

## Conclusion

Host blood mRNA signatures show considerable promise as triage tests for TB. Signatures designed for TB diagnosis meeting at least one TPP minimum performance criterion in independent validation sets containing healthy controls or LTBI populations should be further optimised in populations with ODs. Similarly signatures for TB diagnosis validated in populations with ODs and signatures for prediction of progression from LTBI to TB disease meeting the minimum TPP should be further optimised and validated under field conditions to confirm their accuracy for use as standalone diagnostic or predictive tests for therapeutic decision-making. There is also need for signature discovery in large “real-world” clinically appropriate populations, without spectrum bias, need for head-to-head comparison of signatures and adaptation and implementation towards a POC test.

## Supporting information

S1 Table PRISMA checklist. Checklist according to the PRISMA reporting guidelines.
(DOCX)

S2 Table Gene matrix. A matrix of all identified signatures with their corresponding gene composition.
(XLSX)

S1 File Data extraction form. Form used for data extraction.
(PDF)

S2 File QUADAS-2 form. Modified individual study quality assessment form.
(PDF)

S3 File Methods and results. Manuscript-specific supplementary methods, figures and tables.
(PDF)
