# Supplementary material for: Performance of diagnostic and predictive host blood transcriptomic signatures for Tuberculosis disease: A systematic review and meta-analysis
Source: PLoS One. 2020 Aug 21;15(8):e0237574. doi: 10.1371/journal.pone.0237574 (PMC7442252; doi:10.1371/journal.pone.0237574)
Supplement: S1 File — Form used for data extraction. (PDF) [file pone.0237574.s003.pdf]

# S1_File

## Performance of diagnostic and predictive host blood transcriptomic signatures for tuberculosis disease: a systematic review and meta-analysis.

**Data Extraction Form**

**Reviewer initials** □ HM □ CZZ □ Other________________

**Part A: Study characteristics**

**Author's last name:** ______________________________ **Publication year:** __________________

**Study title:**

__________________________________________________________________________

**Country of study population:** __________________ **Study #**__________________

**TB Burden in study population:** □Low □Intermediate □ High

**Study design:** □Cross-sectional □Cohort □ Case control □ RCT □ Other _______________

**Study type:** □ Diagnostic □ Predictive *if predictive; follow-up time________months*

**Study purpose:** □ Discovery □ Validation

**Sampling:** □ Consecutive □Convenient □Random □Other__________

**Part C: Characteristics of tests**

**Index sample type** : □ Whole blood □ PBMC

**Index test (signature) type** : □mRNA □Other________________________________________

**Signature name** : ________________ **# of genes:** ______

**Signature discovery method:** □ RNA Seq □Micro array □PCR □Other________________

**Signature model** : □ Pairwise □Random forest □SVM □Other________________

**TB disease gold standard** : □Culture □Xpert MTB/RIF □ Smear □ Other_____________

**Part B: Population characteristics**

**Population:** □Adults □Adolescents □ Children □Mixed □ Undefined

**Age range:** ____________

**Participant's cohort (test/validation) and disease status****

| Cohort Type | Healthy Controls[a] | LTBI[b] | Other Diseases[c] | TB Disease[d] | Total Enrolled |
|---|---|---|---|---|---|
| | | | | | |
| | | | | | |
| | | | | | |
| | | | | | |
| | | | | | |
| | | | | | |
| | | | | | |

*** For diagnostic studies, the number of participants in each category represents the numbers at enrolment while for predictive studies; a, b and c represent the number of participants in each category at enrolment that gave rise to the cases (d) at the end of follow-up period.*

S2_File

# S1_File

**Description of negative population**

[a]Healthy Controls: □ Healthy endemics □ Healthy non-endemics □ TB Contacts □ Other, describe:______________________________________________________________

[b]Other diseases: □ Non-respiratory diseases □ Respiratory diseases

[c]LTBI : □TST>5mm □TST>10mm □IGRA □ N/A

*If IGRA, IGRA type : □QFT □Elispot □Other*_______________________

[d]*TB disease: For diagnostic studies, this number represents the number of participants included in the study at enrolment while for predictive studies; this number represents the number of participants diagnosed with TB at the end of follow-up period.*

**Part D: Outcomes**

| Cohort Type | Sens | Spec | AUC | TP | TN | FP | FN | LR+ | LR- | RR |
|---|---|---|---|---|---|---|---|---|---|---|
| | | | | | | | | | | |
| | | | | | | | | | | |
| | | | | | | | | | | |
| | | | | | | | | | | |
| | | | | | | | | | | |
| | | | | | | | | | | |

**Key:** *Sens= Sensitivity; Spec= Specificity; AUC= Area under the curve; TP = True positives; FP = False positives; TN = True negatives; FN = False negatives; LR+ = Positive likelihood ratio; LR- = Negative likelihood ratio; RR = Rate Ratio for predictive studies only.*

**Inclusion/Exclusion Decision**

□Included □Excluded □ Pending

If excluded, reason:

□ Excluded by title

□ Children only

□ Different index test

□ Reference test neither Culture, Xpert/MTB RIF nor Smear Microscopy

□ HIV infected only

□ Mixed HIV infected and uninfected and could not desegregate the participants

□ Mixed children and adults and could not desegregate the participants

□ Other study design

□ Diagnostic performance data not reported and unable to get from authors

S2_File
