# Supplementary material for: Performance of diagnostic and predictive host blood transcriptomic signatures for Tuberculosis disease: A systematic review and meta-analysis
Source: PLoS One. 2020 Aug 21;15(8):e0237574. doi: 10.1371/journal.pone.0237574 (PMC7442252; doi:10.1371/journal.pone.0237574)
Supplement: S2 File — Modified individual study quality assessment form. (PDF) [file pone.0237574.s004.pdf]

Performance of diagnostic and predictive host blood transcriptomic signatures for tuberculosis disease: a systematic review and meta-analysis.

Individual Study Quality Assessment Tool (QUADAS-2)

Study Title: ____

Author: ____________________ **Publication year:** ___________ **Study #**_______

Reviewer initials □ HM □ CZZ □ Other________________

Study type: □ Diagnostic □ Predictive

Domain 1. Patient selection

(a) Risk of bias: L/H/U

- ❖ Was a consecutive or random sample of patients enrolled? Y/N/U
- ❖ Was a case-control design avoided? Y/N/U

(b) Applicability Concerns: L/H/U

- ❖ Is there concern that the included patients do not match the review question? Y/N/U

Domain 2. Index Test (Transcriptomic Signature)

(a) Risk of bias: L/H/U

- ❖ Were the Transcriptomic signature test results interpreted without knowledge of the results of the MTB culture or XPert/MTB RIF or Smear Microscopy? Y/N/U

(b) Applicability Concerns: L/H/U

- ❖ Is there concern that the Transcriptomic signature test, its conduct, or interpretation differ from the review question? Y/N/U

Domain 3. Reference Standard (MTB Culture or Xpert/MTB RIF or Smear Microscopy)

(a) Risk of bias: L/H/U

- ❖ Was MTB Culture or Xpert/MTB RIF or Smear Microscopy used as the reference standard? Y/N/U

(b) Applicability Concerns: L/H/U

- ❖ Is there concern that the MTB Culture or Xpert/MTB RIF or Smear Microscopy, its conduct, or interpretation differ from the review question? Y/N/U

Domain 4. Flow and timing

(a) ***Risk of bias: L/H/U***

- ❖ Did patients in the study receive the same reference standard? Y/N/U
- ❖ Were all patients included in the analysis? Y/N/U

Overall Study Rating L/H/U

For each domain, ‘risk of bias’ or ‘concerns regarding applicability’ will be scored as ‘L’ if all responses in that domain are scored as ‘Y’ and ‘H’ if any of the responses is ‘N’ and ‘U’ if we are unclear for all the responses.

Legend: Y=Yes;N=No;U=Unclear ***H=high;L=Low;U=Unclear***
