# Supplementary material for: Performance of diagnostic and predictive host blood transcriptomic signatures for Tuberculosis disease: A systematic review and meta-analysis
Source: PLoS One. 2020 Aug 21;15(8):e0237574. doi: 10.1371/journal.pone.0237574 (PMC7442252; doi:10.1371/journal.pone.0237574)
Supplement: S3 File — Manuscript-specific supplementary methods, figures and tables. (PDF) [file pone.0237574.s005.pdf]

Performance of diagnostic and predictive host blood transcriptomic signatures for tuberculosis disease: a systematic review and meta-analysis.

Methods:

Quality assessment.

For each of the four domains in QUADAS-2, namely 'patient selection', 'index test', 'reference standard' and 'flow and timing', risk of bias was scored as 'low' if all responses in that domain were answered as 'yes', 'high' if any of the responses were answered as 'no' or 'unclear' and 'unclear' if it was unclear for all the responses. Similarly, we judged "low applicability concerns" in each domain if all signalling questions in that domain were answered as 'yes', "high applicability concerns" if any question was answered as 'no' and "unclear applicability concerns" if answered as such.

For the item "Patient Selection", we considered a prospective or cross-sectional study design with consecutively or randomly enrolled participants as low risk and a case-control design as high risk of bias. For diagnostic studies, we considered a clinically relevant control population (such as individuals with other diseases (OD)) as low "applicability concerns" in patient selection. High "applicability concerns" were for control groups involving household TB contacts (HHC) or individuals with latent TB infection (LTBI). Therefore, a score of "low" for patient selection in studies of TB diagnosis, meant that participants included an OD control group who were consecutively or randomly enrolled in a prospective, cohort or cross-sectional study. For studies of prediction to TB disease, we considered HHC or LTBI individuals as low "applicability concerns" in patient selection. High "applicability concerns" were for populations involving healthy uninfected participants with no known close contact to a person with active TB disease. Therefore, a score of "low" for patient selection in studies of prediction to TB disease meant that the population involved LTBI or HHC participants who were consecutively or randomly enrolled in a prospective, cohort or cross-sectional study. For the "Index Test", we considered whether the signature was interpreted without the knowledge of the reference standard; blinding to index test status was considered low risk of bias. Applicability concerns for the index test were considered low if blinding to index test status done and high if not done. For the "Reference Standard", we considered whether the reference standard included MTB culture, Xpert MTB/RIF, or smear microscopy and therefore likely to classify TB disease correctly. Therefore, a score of ''low'' for risk of bias implied utilisation of MTB culture, Xpert MTB/RIF, or smear microscopy as reference standards. Applicability concerns were low if MTB culture, Xpert MTB/RIF, or smear microscopy were used as a reference standard and high if none of the three reference standards were used. For "Flow and Timing", we considered whether all participants were evaluated using the same reference standard and whether they were all included in the analysis, suggesting low risk of bias and high risk if the opposite happened.

S3_File: **Manuscript-specific supplementary material**

Results

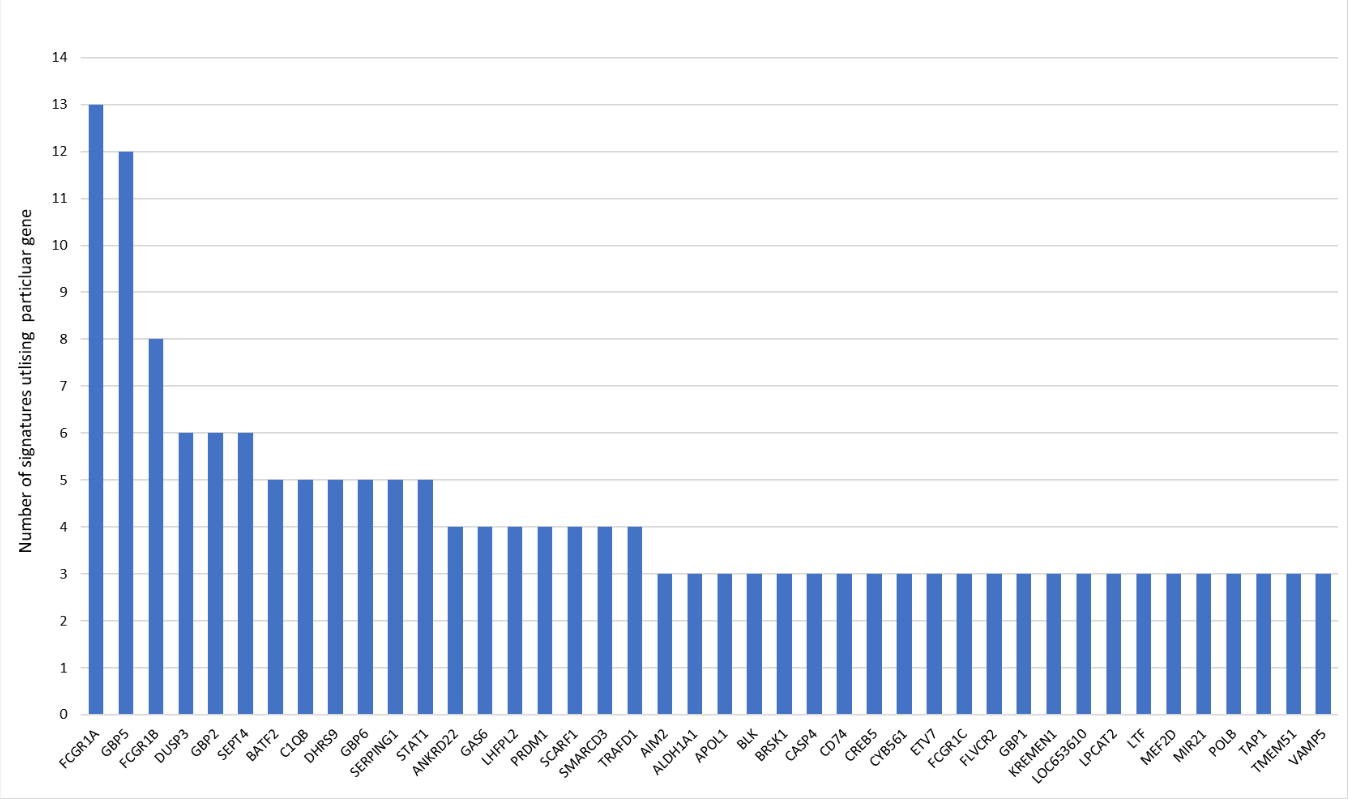

Fig 1. Column chart of forty-two most frequently utilised genes in transcriptomic signatures of either TB diagnosis or prediction to TB disease.

S3_File: Manuscript-specific supplementary material

Study Name	Signature	Patient Selection		Index Test		Reference Standard		Flow & Timing
		Risk of Bias	Applicability Concerns	Risk of Bias	Applicability Concerns	Risk of Bias	Applicability Concerns	Risk of Bias
Berry2010a	Berry393_2010	LR	HR	UC	UC	LR	LR	LR
Berry2010b	Berry86_2010	LR	LR	UC	UC	LR	LR	LR
Bloom2013	Bloom144_2010	HR	LR	UC	UC	LR	LR	LR
daCosta2015a	daCosta2_2015	HR	LR	UC	UC	LR	LR	LR
daCosta2015b	daCosta3_2015	HR	LR	UC	UC	LR	LR	LR
Dawany2015a	Dewany251_2015	HR	HR	UC	UC	LR	LR	LR
Dawany2015b	Dewany251_2015	HR	HR	UC	UC	LR	LR	LR
Dawany2015c	Dewany251_2015	HR	HR	UC	UC	LR	LR	LR
Dawany2015d	Dewany251_2015	HR	HR	UC	UC	LR	LR	LR
Dawany2015e	Dewany251_2015	HR	HR	UC	UC	LR	LR	LR
Dawany2015f	Dewany251_2015	HR	HR	UC	UC	LR	LR	LR
DeAraujo2016a	DeAraujo1_2016	HR	HR	LR	LR	LR	LR	LR
DeAraujo2016b	DeAraujo1_2016	HR	HR	LR	LR	LR	LR	LR
DeAraujo2016c	DeAraujo1_2016	HR	HR	LR	LR	LR	LR	LR
DeAraujo2016d	DeAraujo1_2016	HR	HR	LR	LR	LR	LR	LR
Francisco2017a	Franscisco2_2017	HR	HR	UC	UC	LR	LR	LR
Francisco2017b	Sweeney3_2016	HR	LR	UC	UC	LR	LR	LR
Francisco2017c	Franscisco2_2017	HR	HR	UC	UC	LR	LR	LR
Francisco2017d	Sweeney3_2016	HR	LR	UC	UC	LR	LR	LR
Francisco2017e	Franscisco2_2017	HR	LR	UC	UC	LR	LR	LR
Francisco2017f	Sweeney3_2016	HR	LR	UC	UC	LR	LR	LR
Haung2015a	Huang13_2015	HR	LR	UC	UC	LR	LR	LR
Huang2018	Huang1_2018	HR	HR	UC	UC	UC	UC	UC
Kaforou2013a	Kaforou27_2013	HR	HR	UC	UC	LR	LR	LR
Kaforou2013b	Kaforou44_2013	HR	LR	UC	UC	LR	LR	LR
Kaforou2013c	Berry393_2010	HR	HR	UC	UC	LR	LR	LR
Kaforou2013d	Berry86_2010	HR	LR	UC	UC	LR	LR	LR
Lee2016a	Lee2_2016	HR	HR	UC	UC	LR	LR	LR
Lee2016b	Lee3_2016	HR	HR	UC	UC	LR	LR	LR
Leong2018a	Berry393_2010	LR	HR	UC	UC	LR	LR	LR
Leong2018b	Berry86_2010	LR	HR	UC	UC	LR	LR	LR
Leong2018c	Jacobsen3_2007	LR	HR	UC	UC	LR	LR	LR
Leong2018d	Kaforou27_2013	LR	HR	UC	UC	LR	LR	LR
Leong2018e	Sambarey10_2017	LR	HR	UC	UC	LR	LR	LR
Leong2018f	Sweeney3_2016	LR	HR	UC	UC	LR	LR	LR
Leong2018g	Zak16_2016	LR	HR	UC	UC	LR	LR	LR
Lu2011	Lu3_2011	HR	HR	UC	UC	LR	LR	LR
Pan2017a	Pan2_2017	HR	HR	UC	UC	LR	LR	LR
Pan2017b	Pan2_2017	HR	HR	UC	UC	LR	LR	LR
Sambarey2017a	Sambarey10_2017	HR	HR	UC	UC	LR	LR	LR
Sambarey2017b	Sambarey10_2017	HR	LR	UC	UC	LR	LR	LR
Sambarey2017c	Sambarey10_2017	HR	HR	UC	UC	LR	LR	LR
Sambarey2017d	Sambarey10_2017	HR	LR	UC	UC	LR	LR	LR
Sweeney2016a	Sweeney3_2016	HR	HR	UC	UC	LR	LR	LR
Sweeney2016b	Sweeney3_2016	HR	HR	UC	UC	LR	LR	LR
Sweeney2016c	Sweeney3_2016	HR	HR	UC	UC	LR	LR	LR
Sweeney2016d	Sweeney3_2016	HR	HR	UC	UC	LR	LR	LR
Sweeney2016e	Sweeney3_2016	HR	LR	UC	UC	LR	LR	LR
Walter2016a	Walter47_2016	HR	LR	UC	UC	LR	LR	LR
Walter2016b	Walter51_2016	HR	HR	UC	UC	LR	LR	LR
Walter2016c	Walter119_2016	HR	LR	UC	UC	LR	LR	LR
Walter2016d	Berry393_2010	HR	HR	UC	UC	LR	LR	LR
Walter2016e	Berry86_2010	HR	LR	UC	UC	LR	LR	LR
Walter2016f	Bloom144_2010	HR	LR	UC	UC	LR	LR	LR
Walter2016g	Kaforou27_2013	HR	HR	UC	UC	LR	LR	LR
Walter2016h	Kaforou44_2013	HR	LR	UC	UC	LR	LR	LR
Walter2016i	Kaforou53_2013	HR	LR	UC	UC	LR	LR	LR
Warsinske2018a	Sweeney3_2016	HR	LR	UC	UC	LR	LR	HR
Zak2016a	Zak16_2016	LR	HR	UC	UC	LR	LR	LR
Zak2016b	Zak16_2016	LR	HR	UC	UC	LR	LR	LR
Zak2016c	Zak16_2016	LR	HR	UC	UC	LR	LR	LR
Zak2016d	Zak16_2016	LR	HR	UC	UC	LR	LR	LR
Zak2016e	Zak16_2016	LR	HR	UC	UC	LR	LR	LR
Zak2016f	Zak16_2016	LR	HR	UC	UC	LR	LR	LR
Zak2016g	Zak16_2016	LR	LR	UC	UC	LR	LR	LR
Zak2016h	Zak16_2016	LR	LR	UC	UC	LR	LR	LR
Zak2016i	Zak16_2016	LR	LR	UC	UC	LR	LR	LR
Zak2016j	Zak16_2016	LR	LR	UC	UC	LR	LR	LR
Zak2016k	Zak16_2016	LR	LR	UC	UC	LR	LR	LR
Zak2016l	Zak16_2016	LR	HR	UC	UC	LR	LR	LR

S3_File: Manuscript-specific supplementary material

Fig 2. Methodological quality summary of all diagnostic studies with independent validation cohorts. LR; Low risk, HR; High risk, UC; Unclear

Study Name	Signature	Patient Selection		Index Test		Reference Standard		Flow & Timing
		Risk of Bias	Applicability Concerns	Risk of Bias	Applicability Concerns	Risk of Bias	Applicability Concerns	Risk of Bias
Cai2014a	Cai1_2014	HR	HR	UC	UC	LR	LR	LR
Cai2014b	Cai1_2014	HR	HR	UC	UC	LR	LR	LR
Darboe2018	Darboe11_2018	HR	HR	UC	UC	LR	LR	LR
Leong2018h	Leong24_2018	HR	HR	UC	UC	LR	LR	LR
Lesho2011	Lesho127_2011	HR	HR	UC	UC	LR	LR	LR
Maertzdolf2011a	Maertzdolf3_2011	HR	HR	UC	UC	LR	LR	LR
Maertzdolf2011b	Maertzdolf5_2011	HR	HR	UC	UC	LR	LR	LR
Satproedprai2015	Satproedprai7_2015	HR	HR	UC	UC	LR	LR	LR
Serrano2016	Serrano2_2016	HR	HR	UC	UC	LR	LR	LR
Wu2007	Wu10_2007	HR	HR	UC	UC	LR	LR	LR

Fig 3. Methodological quality summary of all diagnostic studies without independent validation cohorts. LR; Low risk, HR; High risk, UC; Unclear

Study Name	Signature	Patient Selection		Index Test		Reference Standard		Flow & Timing
		Risk of Bias	Applicability Concerns	Risk of Bias	Applicability Concerns	Risk of Bias	Applicability Concerns	Risk of Bias
Roe2019b	Roe3_2019	LR	LR	UC	UC	HR	HR	LR
Suliman2018b	Suliman4_2018	LR	LR	LR	LR	LR	LR	LR
Warsinske2018b	Sweeney3_2016	LR	LR	LR	LR	LR	LR	LR
Zak2016p	Zak16_2016	LR	LR	LR	LR	LR	LR	LR

Fig 4. Methodological quality summary of studies of prediction to TB disease in independent validation cohorts. LR; Low risk, HR; High risk, UC; Unclear

S3_File: Manuscript-specific supplementary material

Study	TP	FP	FN	TN	Comparator	Dataset	Signature	Sensitivity (95% CI)	Specificity (95% CI)
Dawany2015a	6	0	1	10	HC	Berry-GSE19435	Dewany251_2015	0.86 [0.42, 1.00]	1.00 [0.69, 1.00]
Dawany2015f	6	0	1	12	HC	Berry-GSE19435	Dewany251_2015	0.86 [0.42, 1.00]	1.00 [0.74, 1.00]
DeAraujo2016a	28	1	6	23	HC	Berry-GSE19491	DeAraujo1_2016	0.82 [0.65, 0.93]	0.96 [0.79, 1.00]
DeAraujo2016c	8	1	0	17	HC	Maertzdolf-GSE34608	DeAraujo1_2016	1.00 [0.63, 1.00]	0.94 [0.73, 1.00]
DeAraujo2016d	10	2	1	50	HC	Bloom-GSE42826	DeAraujo1_2016	0.91 [0.59, 1.00]	0.96 [0.87, 1.00]
Francisco2017e	112	27	32	182	HC	Not-Publicly-Available	Franscisco2_2017	0.78 [0.70, 0.84]	0.87 [0.82, 0.91]
Francisco2017f	123	61	21	148	HC	Not-Publicly-Available	Sweeney3_2016	0.85 [0.79, 0.91]	0.71 [0.64, 0.77]
Huang2018	83	9	32	81	HC	Not-Publicly-Available	Huang1_2018	0.72 [0.63, 0.80]	0.90 [0.82, 0.95]
Pan2017a	56	9	10	77	HC	Not-Publicly-Available	Pan2_2017	0.85 [0.74, 0.92]	0.90 [0.81, 0.95]
Sweeney2016a	39	7	7	30	HC	Maertzdolf-GSE28623	Sweeney3_2016	0.85 [0.71, 0.94]	0.81 [0.65, 0.92]
Sweeney2016b	8	0	0	18	HC	Maertzdolf-GSE34608	Sweeney3_2016	1.00 [0.63, 1.00]	1.00 [0.81, 1.00]
Sweeney2016c	11	6	7	12	HC	Ottenhoff-GSE56153	Sweeney3_2016	0.61 [0.36, 0.83]	0.67 [0.41, 0.87]
Zak2016c	19	4	2	8	HC	Berry-GSE19444	Zak16_2016	0.90 [0.70, 0.99]	0.67 [0.35, 0.90]
Zak2016f	32	2	3	111	HC	Bloom-GSE42825/26/30	Zak16_2016	0.91 [0.77, 0.98]	0.98 [0.94, 1.00]
Berry2010a	19	1	1	30	LTBI	Berry-GSE19491	Berry393_2010	0.95 [0.75, 1.00]	0.97 [0.83, 1.00]
Dawany2015b	25	3	4	35	LTBI	Bloom-GSE40553	Dewany251_2015	0.86 [0.68, 0.96]	0.92 [0.79, 0.98]
Dawany2015e	18	3	2	28	LTBI	Berry-GSE19444	Dewany251_2015	0.90 [0.68, 0.99]	0.90 [0.74, 0.98]
DeAraujo2016b	17	1	3	30	LTBI	Berry-GSE19491	DeAraujo1_2016	0.85 [0.62, 0.97]	0.97 [0.83, 1.00]
Kaforou2013a	19	2	1	29	LTBI	Berry-GSE19491	Kaforou27_2013	0.95 [0.75, 1.00]	0.94 [0.79, 0.99]
Kaforou2013c	85	13	12	70	LTBI	Kaforou-GSE37250	Berry393_2010	0.88 [0.79, 0.93]	0.84 [0.75, 0.91]
Leong2018a	26	1	2	15	LTBI	Leong-GSE101705	Berry393_2010	0.93 [0.76, 0.99]	0.94 [0.70, 1.00]
Leong2018b	26	1	2	15	LTBI	Leong-GSE101705	Berry86_2010	0.93 [0.76, 0.99]	0.94 [0.70, 1.00]
Leong2018c	24	2	4	14	LTBI	Leong-GSE101705	Jacobsen3_2007	0.86 [0.67, 0.96]	0.88 [0.62, 0.98]
Leong2018d	26	1	2	15	LTBI	Leong-GSE101705	Kaforou27_2013	0.93 [0.76, 0.99]	0.94 [0.70, 1.00]
Leong2018e	25	1	3	15	LTBI	Leong-GSE101705	Sambarey10_2017	0.89 [0.72, 0.98]	0.94 [0.70, 1.00]
Leong2018f	24	3	4	13	LTBI	Leong-GSE101705	Sweeney3_2016	0.86 [0.67, 0.96]	0.81 [0.54, 0.96]
Leong2018g	26	1	2	15	LTBI	Leong-GSE101705	Zak16_2016	0.93 [0.76, 0.99]	0.94 [0.70, 1.00]
Lu2011	12	2	5	17	LTBI	Not-Publicly-Available	Lu3_2011	0.71 [0.44, 0.90]	0.89 [0.67, 0.99]
Pan2017b	47	5	19	73	LTBI	Not-Publicly-Available	Pan2_2017	0.71 [0.59, 0.82]	0.94 [0.86, 0.98]
Sambarey2017a	42	3	9	33	LTBI	Kaforou-GSE37250	Sambarey10_2017	0.82 [0.69, 0.92]	0.92 [0.78, 0.98]
Sambarey2017c	37	4	10	43	LTBI	Kaforou-GSE37250	Sambarey10_2017	0.79 [0.64, 0.89]	0.91 [0.80, 0.98]
Sweeney2016d	40	4	6	21	LTBI	Maertzdolf-GSE28623	Sweeney3_2016	0.87 [0.74, 0.95]	0.84 [0.64, 0.95]
Walter2016b	88	11	9	72	LTBI	Kaforou-GSE37250	Walter51_2016	0.91 [0.83, 0.96]	0.87 [0.78, 0.93]
Walter2016d	31	2	4	33	LTBI	Walter-GSE73408	Berry393_2010	0.89 [0.73, 0.97]	0.94 [0.81, 0.99]
Walter2016g	33	3	2	32	LTBI	Walter-GSE73408	Kaforou27_2013	0.94 [0.81, 0.99]	0.91 [0.77, 0.98]
Zak2016a	19	10	2	11	LTBI	Berry-GSE19444	Zak16_2016	0.90 [0.70, 0.99]	0.52 [0.30, 0.74]
Zak2016b	18	0	2	30	LTBI	Berry-GSE19442	Zak16_2016	0.90 [0.68, 0.99]	1.00 [0.88, 1.00]
Zak2016d	46	10	5	25	LTBI	Kaforou-GSE37250	Zak16_2016	0.90 [0.79, 0.97]	0.71 [0.54, 0.85]
Zak2016e	42	3	4	45	LTBI	Kaforou-GSE37250	Zak16_2016	0.91 [0.79, 0.98]	0.94 [0.83, 0.99]
Zak2016l	26	3	3	35	LTBI	Bloom-GSE40553	Zak16_2016	0.90 [0.73, 0.98]	0.92 [0.79, 0.98]
Dawany2015c	9	0	4	29	LTBI+HC	Berry-GSE19439	Dewany251_2015	0.69 [0.39, 0.91]	1.00 [0.88, 1.00]
Dawany2015d	16	1	5	32	LTBI+HC	Berry-GSE19444	Dewany251_2015	0.76 [0.53, 0.92]	0.97 [0.84, 1.00]
Lee2016a	14	0	1	32	LTBI+HC	Not-Publicly-Available	Lee2_2016	0.93 [0.68, 1.00]	1.00 [0.89, 1.00]
Lee2016b	15	1	0	30	LTBI+HC	Not-Publicly-Available	Lee3_2016	1.00 [0.78, 1.00]	0.97 [0.83, 1.00]
Berry2010b	18	16	2	80	OD	Berry-GSE19491	Berry86_2010	0.90 [0.68, 0.99]	0.83 [0.74, 0.90]
daCosta2015a	33	3	2	16	OD	Bloom-GSE42834	daCosta2_2015	0.94 [0.81, 0.99]	0.84 [0.60, 0.97]
daCosta2015b	33	3	2	16	OD	Bloom-GSE42834	daCosta3_2015	0.94 [0.81, 0.99]	0.84 [0.60, 0.97]
Francisco2017a	138	31	6	178	OD	Not-Publicly-Available	Franscisco2_2017	0.96 [0.91, 0.98]	0.85 [0.80, 0.90]
Francisco2017b	107	19	37	190	OD	Not-Publicly-Available	Sweeney3_2016	0.74 [0.66, 0.81]	0.91 [0.86, 0.94]
Francisco2017c	215	197	60	93	OD	Not-Publicly-Available	Franscisco2_2017	0.78 [0.73, 0.83]	0.32 [0.27, 0.38]
Francisco2017d	169	154	106	136	OD	Not-Publicly-Available	Sweeney3_2016	0.61 [0.55, 0.67]	0.47 [0.41, 0.53]
Kaforou2013b	20	3	0	80	OD	Kaforou-GSE37250	Kaforou44_2013	1.00 [0.83, 1.00]	0.96 [0.90, 0.99]
Kaforou2013d	69	20	28	63	OD	Kaforou-GSE37250	Berry86_2010	0.71 [0.61, 0.80]	0.76 [0.65, 0.85]
Sambarey2017b	47	18	4	16	OD	Kaforou-GSE37250	Sambarey10_2017	0.92 [0.81, 0.98]	0.47 [0.30, 0.65]
Sambarey2017d	32	10	15	39	OD	Kaforou-GSE37250	Sambarey10_2017	0.68 [0.53, 0.81]	0.80 [0.66, 0.90]
Sweeney2016e	4	7	4	11	OD	Maertzdolf-GSE34608	Sweeney3_2016	0.50 [0.16, 0.84]	0.61 [0.36, 0.83]
Walter2016a	87	19	10	64	OD	Kaforou-GSE37250	Walter47_2016	0.90 [0.82, 0.95]	0.77 [0.67, 0.86]
Walter2016e	32	10	3	29	OD	Walter-GSE73408	Berry86_2010	0.91 [0.77, 0.98]	0.74 [0.58, 0.87]
Walter2016h	24	8	11	31	OD	Walter-GSE73408	Kaforou44_2013	0.69 [0.51, 0.83]	0.79 [0.64, 0.91]
Zak2016g	32	2	3	14	OD	Bloom-GSE42825/26/30	Zak16_2016	0.91 [0.77, 0.98]	0.88 [0.62, 0.98]
Zak2016h	31	1	4	13	OD	Bloom-GSE42825/26/30	Zak16_2016	0.89 [0.73, 0.97]	0.93 [0.66, 1.00]
Zak2016i	32	23	3	38	OD	Bloom-GSE42825/26/30	Zak16_2016	0.91 [0.77, 0.98]	0.62 [0.49, 0.74]
Zak2016j	46	18	5	16	OD	Kaforou-GSE37250	Zak16_2016	0.90 [0.79, 0.97]	0.47 [0.30, 0.65]
Zak2016k	41	11	5	38	OD	Kaforou-GSE37250	Zak16_2016	0.89 [0.76, 0.96]	0.78 [0.63, 0.88]
Bloom2013	7	3	1	31	OD+HC	Bloom-GSE42834	Bloom144_2010	0.88 [0.47, 1.00]	0.91 [0.76, 0.98]
Huang2015a	8	2	0	32	OD+HC	Bloom-GSE42825	Huang13_2015	1.00 [0.63, 1.00]	0.94 [0.80, 0.99]
Warsinske2018a	158	56	18	131	OD+HC+LTBI	Zak-GSE79362	Sweeney3_2016	0.90 [0.84, 0.94]	0.70 [0.63, 0.77]
Walter2016c	103	29	14	117	OD+LTBI	Kaforou-GSE37250	Walter119_2016	0.88 [0.81, 0.93]	0.80 [0.73, 0.86]
Walter2016f	30	17	5	57	OD+LTBI	Walter-GSE73408	Bloom144_2010	0.86 [0.70, 0.95]	0.77 [0.66, 0.86]
Walter2016i	27	18	8	56	OD+LTBI	Walter-GSE73408	Kaforou53_2013	0.77 [0.60, 0.90]	0.76 [0.64, 0.85]

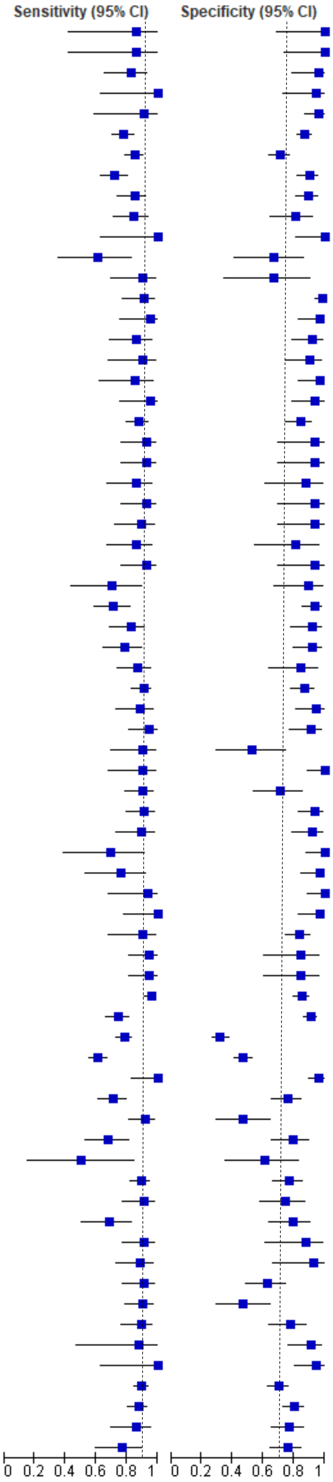

Fig 5. Forest plots of sensitivity and specificity of mRNA transcriptomic signatures for diagnosis of TB disease in independent validation cohorts (all studies). *HC; Healthy Controls, LTBI; Latent TB infection, OD; Other diseases. Each signature validated in a different population is represented as a separate entry. Vertical dotted lines correspond to 90% sensitivity and 70% specificity*

S3_File: Manuscript-specific supplementary material

Study	TP	FP	FN	TN	Comparator	Dataset	Signature	Sensitivity (95% CI)	Specificity (95% CI)
Cai2014a	134	5	28	40	HC	Cai-GSE54992	Cai1_2014	0.83 [0.76, 0.88]	0.89 [0.76, 0.96]
Cai2014b	122	29	43	133	LTBI	Cai-GSE54992	Cai1_2014	0.74 [0.67, 0.80]	0.82 [0.75, 0.88]
Darboe2018	30	6	0	24	LTBI	Not-Publicly-Available	Darboe11_2018	1.00 [0.88, 1.00]	0.80 [0.61, 0.92]
Leong2018h	26	1	2	15	LTBI	Leong-GSE101705	Leong24_2018	0.93 [0.76, 0.99]	0.94 [0.70, 1.00]
Lesho2011	5	0	0	18	LTBI+HC	Not-Publicly-Available	Lesho127_2011	1.00 [0.48, 1.00]	1.00 [0.81, 1.00]
Maertzdorf2011a	28	3	4	31	LTBI	Maertzdolf-GSE25534	Maertzdorf3_2011	0.88 [0.71, 0.96]	0.91 [0.76, 0.98]
Maertzdorf2011b	30	1	2	33	LTBI	Maertzdolf-GSE25534	Maertzdorf5_2011	0.94 [0.79, 0.99]	0.97 [0.85, 1.00]
Satproedprai2015	33	0	7	56	OD+HC+LTBI	Berry-GSE19491	Satproedprai7_2015	0.82 [0.67, 0.93]	1.00 [0.94, 1.00]
Serrano2016	9	4	1	16	LTBI+HC	Not-Publicly-Available	Serano2_2016	0.90 [0.55, 1.00]	0.80 [0.56, 0.94]
Wu2007	16	1	14	23	LTBI	Not-Publicly-Available	Wu10_2007	0.53 [0.34, 0.72]	0.96 [0.79, 1.00]

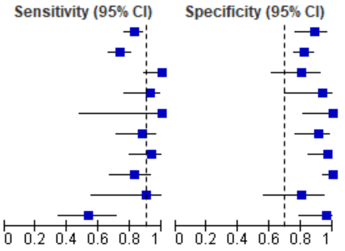

Fig 6. Forest plots of sensitivity and specificity of mRNA transcriptomic signatures for diagnosis of TB disease for studies without independent validation cohorts. *HC; Healthy Controls, LTBI; Latent TB infection, OD; Other diseases. Each signature validated in a different population is represented as a separate entry. Vertical dotted lines correspond to 90% sensitivity and 70% specificity*

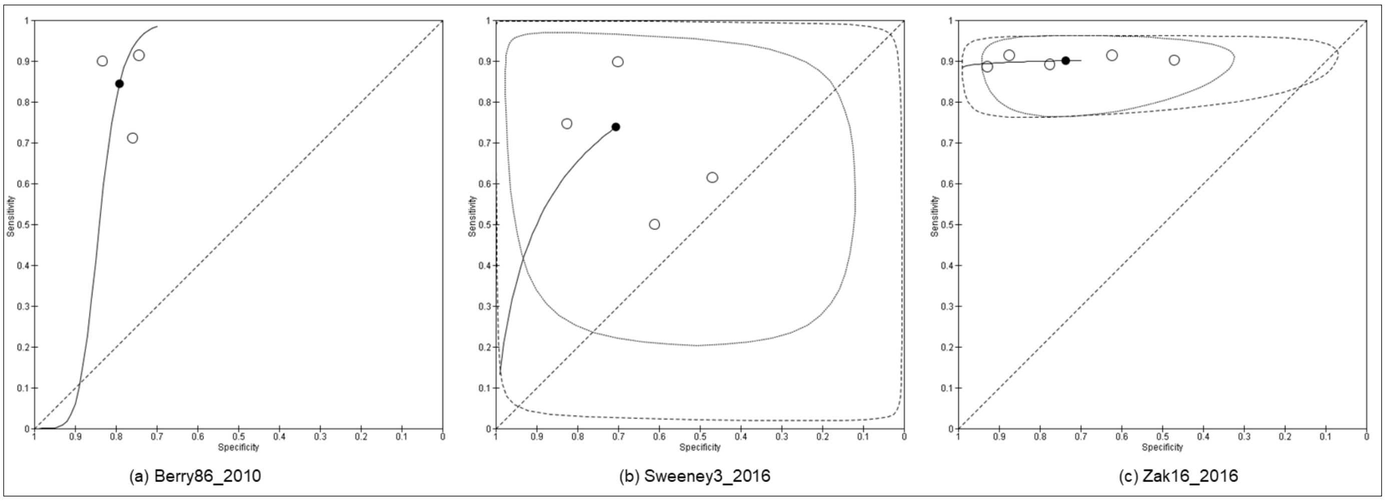

Fig 7. Summary receiver operating characteristic (SROC) curves for the Berry86_2010, Sweeney3_2016 and Zak16_2016 signatures. *Black dot is the summary estimate, white circles are individual study estimates, solid line passing through summary estimate is SROC curve. Solid line around estimates is 95% confidence region, dashed line around estimates is 95% prediction region*

Outcome	Study design	# Studies (Sample size)	Risk of bias (Study limitations)	Inconsistence	Indirectness	Imprecision	Publication bias	Final quality	Effect per 100,000	Importance[1]
True Positives	Cohort, Case-Control, Cross-Section	70 (7,765)	Very serious[2] (-2)	Very serious[3] (-2)	No serious indirectness[4]	Serious[5] (-1)	Likely[6]	Very low ⊕OOO	Prevalence[7] 2% : 1,780	Critical
True Negative	Cohort, Case-Control, Cross-Section	70 (7,765)	Very serious[2] (-2)	Very serious[3] (-2)	No serious indirectness[4]	Serious[5] (-1)	Likely[6]	Very low ⊕OOO	Prevalence[7] 2% : 88,200	Critical
False Positives	Cohort, Case-Control, Cross-Section	70 (7,765)	Very serious[2] (-2)	Very serious[3] (-2)	No serious indirectness[4]	Serious[5] (-1)	Likely[6]	Very low ⊕OOO	Prevalence[7] 2% : 9,800	Critical
False Negatives	Cohort, Case-Control, Cross-Section	70 (7,765)	Very serious[2] (-2)	Very serious[3] (-2)	No serious indirectness[4]	Serious[5] (-1)	Likely[6]	Very low ⊕OOO	Prevalence[7] 2% : 220	Critical

1. The Importance of outcomes was classified as either "Critial", "Important" or "Not important" according to their relative importance
2. Majority of studies were case-controls and lacked both a representative patient spectrum and blinding
3. Substantial heterogeneity was observed in the study results
4. No downgrade was applied for indirectness though diagnostic accuracy is considered a surrogate for paitent-important outcomes.
5. Diagnostic accuracy estimates were not pooled. There was a considerable number of studies with wide 95% CI. We down-graded by one point only, as there were a large number of studies and we had already down-graded for inconsistency
6. We did not down-grade for publication bias. The data in this systematic review did not allow for formal assessment of publication bias with methods such as funnel plots or regression analysis. Publication bias can not be ruled out as studies may not have been published in which mRNA signatures showed poor diagnostic accuracy for TB
7. What is the meaning of these results among people being screened for TB disease, given a 2% prevalance of disease

Explanation: Based on a sample size of 7,765, median sensitity=89.5% and median specificity=90%, we rated the quality of the evidence as high when no points were subtracted, moderate when only one point was subtracted, low when two points were subtracted and very low when more than two points were subtracted. Deduction of points was based on the five factors that decrease study quality; study limitations, incosistence in results accross studies, indirectness in evidence, imprecision in summary estimates, and possibility of reporting bias. For each outcome, evidence from cohort and cross-sectional studies started as high quality while that from case-control studies started as moderate quality. We deducted two, one and zero points for "very serious", "serious", and "no serious" issues identified respectively, and the deducted points are shown in brackets. Reporting bias was classfied as either "very likely", "likely" and "not likely".

Figure 8. GRADE evidence profile: mRNA signatures for the diagnosis of TB (all studies).

Table 1. Characteristics of studies for diagnosis of TB included in the systematic review.

Ref	Study Name	Study Entry ID	Signature Name	Biomarker Description	Dataset	Signature discovery model *	Control Type	TB	Controls	Sens [95% CI]	Spec [95% CI]	AUC	Validation Set	Index Sample	TB reference standard	Population	Sample Country
23	Berry 2010	Berry2010a	Berry393_2010	393 transcript signature	Berry-GSE19491	K nearest neighbour	LTBI	20	31	0.95 [0.75, 1.00]	0.97 [0.83, 1.00]	NR	Y	Whole Blood	Culture	Adults	South Africa, Malawi
		Berry2010b	Berry86_2010	86 transcript signature	Berry-GSE19491		OD	20	96	0.90 [0.68, 0.99]	0.83 [0.74, 0.90]	NR	Y				
38	Bloom 2013	Bloom2013	Bloom144_2010	144 transcript signature	Bloom-GSE42834	Support vector machine	OD+HC	8	34	0.88 [0.47, 1.00]	0.91 [0.76, 0.98]	NR	Y	Whole Blood	Culture	Adults	South Africa
49	DaCosta 2015	DaCosta2015a	daCosta2_2015	GBP5; FCGR1A	Bloom-GSE42834	Random forest	OD	35	19	0.94 [0.81, 0.99]	0.84 [0.60, 0.97]	0.96	Y	Whole Blood	Culture	Adults	South Africa
		DaCosta2015b	daCosta3_2015	GBP5; FCGR1A; GZMA	Bloom-GSE42834		OD	35	19	0.94 [0.81, 0.99]	0.84 [0.60, 0.97]	0.96	Y				
42	Dawany 2015	Dawany2015a	Dewany251_2015	251 transcript signature	Berry-GSE19435	Support vector machine	HC	7	10	0.86 [0.42, 1.00]	1.00 [0.69, 1.00]	1.00	Y	Whole Blood	Culture	Adults	UK
		Dawany2015b	Dewany251_2015		Bloom-GSE40553		LTBI	29	38	0.86 [0.68, 0.96]	0.92 [0.79, 0.98]	0.97	Y				South Africa
		Dawany2015c	Dewany251_2015		Berry-GSE19439		LTBI+HC	13	29	0.69 [0.39, 0.91]	1.00 [0.88, 1.00]	0.94	Y				UK
		Dawany2015d	Dewany251_2015		Berry-GSE19444		LTBI+HC	21	33	0.76 [0.53, 0.92]	0.97 [0.84, 1.00]	0.89	Y				UK
		Dawany2015e	Dewany251_2015		Berry-GSE19442		LTBI	20	31	0.90 [0.68, 0.99]	0.90 [0.74, 0.98]	0.92	Y				South Africa

		Dawany2015f	Dewany251_2015		Berry-GSE19435		HC	7	12	0.86 [0.42, 1.00]	1.00 [0.74, 1.00]	0.98	Y				UK
43	DeAraujo 2016	DeAraujo2016a	DeAraujo1_2016	NPC2	Berry-GSE19491	Classification tree	HC	34	24	0.82 [0.65, 0.93]	0.96 [0.79, 1.00]	0.95	Y	Whole Blood	Culture	Adults	UK
		DeAraujo2016b	DeAraujo1_2016	NPC2	Berry-GSE19491		LTBI	20	31	0.85 [0.62, 0.97]	0.97 [0.83, 1.00]	0.97	Y				South Africa
		DeAraujo2016c	DeAraujo1_2016	NPC2	Maertzdolf-GSE34608		HC	8	18	1.00 [0.63, 1.00]	0.94 [0.73, 1.00]	0.99	Y				Germany
		DeAraujo2016d	DeAraujo1_2016	NPC2	Bloom-GSE42826		HC	11	52	0.91 [0.59, 1.00]	0.96 [0.87, 1.00]	0.99	Y				UK, France
44	Francisco 2017	Francisco2017a	Franscisco2_2017	GBP5; KLF2	NR	Random forest	OD	144	209	0.96 [0.91, 0.98]	0.85 [0.80, 0.90]	0.89	Y	Whole Blood	Culture & Smear	Adults	China
		Francisco2017b	Sweeney3_2016	GBP5; DUSP3; KLF2	NR		OD	144	209	0.74 [0.66, 0.81]	0.91 [0.86, 0.94]	0.71	Y	Whole Blood			
		Francisco2017c	Franscisco2_2017	GBP5; KLF2	NR		OD	275	290	0.78 [0.73, 0.83]	0.32 [0.27, 0.38]	0.54	Y	PBMC			
		Francisco2017d	Sweeney3_2016	GBP5; DUSP3; KLF2	NR		OD	275	290	0.61 [0.55, 0.67]	0.47 [0.41, 0.53]	0.53	Y	PBMC			
		Francisco2017e	Franscisco2_2017	GBP5; KLF2	NR		HC	144	209	0.78 [0.70, 0.84]	0.87 [0.82, 0.91]	0.86	Y	Whole Blood			
		Francisco2017f	Sweeney3_2016	GBP5; DUSP3; KLF2	NR		HC	144	209	0.85 [0.79, 0.91]	0.71 [0.64, 0.77]	0.85	Y	Whole Blood			
45	Haung 2015	Huang2015a	Huang13_2015	13 transcript signature	Bloom-GSE42825	Support vector machine	OD+HC	8	34	1.00 [0.63, 1.00]	0.94 [0.80, 0.99]	NR	Y	PBMC	Culture	Adults	UK, France

46	Huang 2018	Huang2018	Huang1_2018	hsa_circRNA_001937	NR	Hierarchical clustering	HC	115	90	0.72 [0.63, 0.80]	0.90 [0.82, 0.95]	0.85	Y	PBMC	Microbiological	Adults	China
24	Kaforou 2013	Kaforou2013a	Kaforou27_2013	27 transcript signature	Berry-GSE19491	Difference of means	LTBI	20	31	0.95 [0.75, 1.00]	0.94 [0.79, 0.99]	0.99	Y	Whole Blood	Culture	Adults	South Africa, Malawi
		Kaforou2013b	Kaforou44_2013	44 transcript signature	Kaforou-GSE37250		OD	20	83	1.00 [0.83, 1.00]	0.96 [0.90, 0.99]	1.00	Y				
		Kaforou2013c	Berry393_2010	393 transcript signature	Kaforou-GSE37250		LTBI	97	83	0.88 [0.79, 0.93]	0.84 [0.75, 0.91]	0.94	Y				
		Kaforou2013d	Berry86_2010	86 transcript signature	Kaforou-GSE37250		OD	97	83	0.71 [0.61, 0.80]	0.76 [0.65, 0.85]	0.78	Y				
50	Lee 2016	Lee2016a	Lee2_2016	PTPRC; ASUN	NR	Naïve Bayes	LTBI+HC	15	32	0.93 [0.68, 1.00]	1.00 [0.89, 1.00]	0.94	Y	PBMC	Smear	Adults	Taiwan
		Lee2016b	Lee3_2016	PTPRC; ASUN; DHX29	NR		LTBI+HC	15	31	1.00 [0.78, 1.00]	0.97 [0.83, 1.00]	0.98	Y	PBMC			
51	Leong 2018	Leong2018a	Berry393_2010	393 transcript signature	Leong-GSE101705	Ridge logistic regression	LTBI	28	16	0.93 [0.76, 0.99]	0.94 [0.70, 1.00]	0.99	Y	Whole Blood	Culture	Adults	India
		Leong2018b	Berry86_2010	86 transcript signature	Leong-GSE101705		LTBI	28	16	0.93 [0.76, 0.99]	0.94 [0.70, 1.00]	0.97	Y				
		Leong2018c	Jacobsen3_2007	RAB33A; FCGR1A; LTF	Leong-GSE101705		LTBI	28	16	0.86 [0.67, 0.96]	0.88 [0.62, 0.98]	0.90	Y				
		Leong2018d	Kaforou27_2013	27 transcript signature	Leong-GSE101705		LTBI	28	16	0.93 [0.76, 0.99]	0.94 [0.70, 1.00]	0.96	Y				
		Leong2018e	Sambarey10_2017	IFI44L; CYP4F3; FCGR1A; TIMM10; BCL6; HK3;	Leong-GSE101705		LTBI	28	16	0.89 [0.72, 0.98]	0.94 [0.70, 1.00]	0.96	Y				

				SMARCD3; RBBP8; RAB13; SLP1													
		Leong2018f	Sweeney3_2016	GBP5; DUSP3; KLF2	Leong-GSE101705		LTBI	28	16	0.86 [0.67, 0.96]	0.81 [0.54, 0.96]	0.90	Y				
		Leong2018g	Zak16_2016	16 transcript signature	Leong-GSE101705		LTBI	28	16	0.93 [0.76, 0.99]	0.94 [0.70, 1.00]	0.96	Y				
		Leong2018h	Leong24_2018	24 transcript signature	Leong-GSE101705		LTBI	28	16	0.93 [0.76, 0.99]	0.94 [0.70, 1.00]	0.98	N				
52	Lu 2011	Lu2011	Lu3_2011	CXCL10; ATP10A; TLR6	NR	Decision tree	LTBI	17	19	0.71 [0.44, 0.90]	0.89 [0.67, 0.99]	NR	Y	PBMC	Culture	Adolescents and Adults	China
54	Pan 2017	Pan2017a	Pan2_2017	RETN; KLK1	NR	Pairwise comparison	HC	66	86	0.85 [0.74, 0.92]	0.90 [0.81, 0.95]	0.94	Y	PBMC	Culture or 2 Smears	Adults	China
		Pan2017b	Pan2_2017	RETN; KLK1	NR		LTBI	66	78	0.71 [0.59, 0.82]	0.94 [0.86, 0.98]	0.92	Y				
56	Sambarey 2017	Sambarey2017a	Sambarey10_2017	I0 transcript signature (IFI44L; CYP4F3; FCGR1A; TIMM10; BCL6; HK3; SMARCD3; RBBP8; RAB13; SLPI)	Kaforou-GSE37250	Linear-discriminant analysis	LTBI	51	36	0.82 [0.69, 0.92]	0.92 [0.78, 0.98]	NR	Y	Whole Blood	Culture	Adults	Malawi
		Sambarey2017b	Sambarey10_2017		Kaforou-GSE37250		OD	51	34	0.92 [0.81, 0.98]	0.47 [0.30, 0.65]	NR	Y				Malawi
		Sambarey2017c	Sambarey10_2017		Kaforou-GSE37250		LTBI	47	47	0.79 [0.64, 0.89]	0.91 [0.80, 0.98]	NR	Y				South Africa
		Sambarey2017d	Sambarey10_2017		Kaforou-GSE37250		OD	47	49	0.68 [0.53, 0.81]	0.80 [0.66, 0.90]	NR	Y				South Africa
58	Sweeney 2016	Sweeney2016a	Sweeney3_2016		Maertzdolf-GSE28623	Forward search	HC	46	37	0.85 [0.71, 0.94]	0.81 [0.65, 0.92]	NR	Y	Whole Blood	Smear + CXR	Adults	Gambia

		Sweeney2016b	Sweeney3_2016	GBP5; DUSP3; KLF2	Maertzdolf-GSE34608		HC	8	18	1.00 [0.63, 1.00]	1.00 [0.81, 1.00]	NR	Y		Culture	Adults	Germany
		Sweeney2016c	Sweeney3_2016		Ottenhoff-GSE56153		HC	18	18	0.61 [0.36, 0.83]	0.67 [0.41, 0.87]	NR	Y		Smear	Adolescents and adults	Indonesia
		Sweeney2016d	Sweeney3_2016		Maertzdolf-GSE28623		LTBI	46	25	0.87 [0.74, 0.95]	0.84 [0.64, 0.95]	NR	Y		Smear + CXR	Adults	Gambia
		Sweeney2016e	Sweeney3_2016		Maertzdolf-GSE34608		OD	8	18	0.50 [0.16, 0.84]	0.61 [0.36, 0.83]	NR	Y		Culture	Adults	Germany
59	Walter 2016	Walter2016a	Walter47_2016	47 transcript signature	Kaforou-GSE37250	Support vector machine	OD	97	83	0.90 [0.82, 0.95]	0.77 [0.67, 0.86]	0.91	Y	Whole Blood	Culture	Adults	South Africa, Malawi
		Walter2016b	Walter51_2016	51 transcript signature	Kaforou-GSE37250		LTBI	97	83	0.91 [0.83, 0.96]	0.87 [0.78, 0.93]	0.95	Y				
		Walter2016c	Walter119_2016	119 transcript signature	Kaforou-GSE37250		OD+LTBI	117	146	0.88 [0.81, 0.93]	0.80 [0.73, 0.86]	0.91	Y				
		Walter2016d	Berry393_2010	393 transcript signature	Walter-GSE73408		LTBI	35	35	0.89 [0.73, 0.97]	0.94 [0.81, 0.99]	0.94	Y	Whole Blood	Culture + Smear	Adults	USA
		Walter2016e	Berry86_2010	86 transcript signature	Walter-GSE73408		OD	35	39	0.91 [0.77, 0.98]	0.74 [0.58, 0.87]	0.90	Y				
		Walter2016f	Bloom144_2010	144 transcript signature	Walter-GSE73408		OD+LTBI	35	74	0.86 [0.70, 0.95]	0.77 [0.66, 0.86]	0.91	Y				
		Walter2016g	Kaforou27_2013	27 transcript signature	Walter-GSE73408		LTBI	35	35	0.94 [0.81, 0.99]	0.91 [0.77, 0.98]	0.98	Y				
		Walter2016h	Kaforou44_2013	44 transcript signature	Walter-GSE73408		OD	35	39	0.69 [0.51, 0.83]	0.79 [0.64, 0.91]	0.83	Y				

		Walter2016i	Kaforou53_2013	53 transcript signature	Walter-GSE73408		OD+LTBI	35	74	0.77 [0.60, 0.90]	0.76 [0.64, 0.85]	0.83	Y				
60	Warsinske 2018	Warsinske2018a	Sweeney3_2016	GBP5; DUSP3; KLF2	Zak-GSE79362	Forward search	OD+HC+ LTBI	17 6	187	0.90 [0.84, 0.94]	0.70 [0.63, 0.77]	NR	Y	Whole Blood	Culture	Adolescen ts and adults	China
25	Zak 2016	Zak2016a	Zak16_2016	16 transcript signature (GBP2; FCGR1A; FCGR1B; GBP5; STAT1; SERPING1; ANKRD22; BATF2; GBP1; APOL1; TRAFD1; SCARF1; SEPT4; TAP1; ETV7; GPB4)	Berry-GSE19444	Support vector machine	LTBI	21	21	0.90 [0.70, 0.99]	0.52 [0.30, 0.74]	0.86	Y	Whole Blood	Culture	Adults	UK
		Zak2016b	Zak16_2016		Berry-GSE19442		LTBI	20	30	0.90 [0.68, 0.99]	1.00 [0.88, 1.00]	0.99	Y				South Africa
		Zak2016c	Zak16_2016		Berry-GSE19444		HC	21	12	0.90 [0.70, 0.99]	0.67 [0.35, 0.90]	0.91	Y				UK
		Zak2016d	Zak16_2016		Kaforou-GSE37250		LTBI	51	35	0.90 [0.79, 0.97]	0.71 [0.54, 0.85]	0.91	Y				Malawi
		Zak2016e	Zak16_2016		Kaforou-GSE37250		LTBI	46	48	0.91 [0.79, 0.98]	0.94 [0.83, 0.99]	0.94	Y				SA
		Zak2016f	Zak16_2016		Bloom-GSE42825/ 26/30		HC	35	113	0.91 [0.77, 0.98]	0.98 [0.94, 1.00]	0.99	Y				UK, France
		Zak2016g	Zak16_2016		Bloom-GSE42825/ 26/30		OD	35	16	0.91 [0.77, 0.98]	0.88 [0.62, 0.98]	0.95	Y				UK, France
		Zak2016h	Zak16_2016		Bloom-GSE42825/ 26/30		OD	35	14	0.89 [0.73, 0.97]	0.93 [0.66, 1.00]	0.91	Y				UK, France
		Zak2016i	Zak16_2016		Bloom-GSE42825/ 26/30		OD	35	61	0.91 [0.77, 0.98]	0.62 [0.49, 0.74]	0.83	Y				UK, France
		Zak2016j	Zak16_2016		Kaforou-GSE37250		OD	51	34	0.90 [0.79, 0.97]	0.47 [0.30, 0.65]	0.74	Y				Malawi

		Zak2016k	Zak16_2016		Kaforou-GSE37250		OD	46	49	0.89 [0.76, 0.96]	0.78 [0.63, 0.88]	0.83	Y				South Africa
		Zak2016l	Zak16_2016		Bloom-GSE40553		LTBI	29	38	0.90 [0.73, 0.98]	0.92 [0.79, 0.98]	0.98	Y				UK, France
40	Cai 2014	Cai2014a	Cai1_2014	C1QC	Cai-GSE54992	Support vector machine	HC	162	45	0.83 [0.76, 0.88]	0.89 [0.76, 0.96]	0.93	N	PBMC	Culture	Adults	China
		Cai2014b	Cai1_2014		Cai-GSE54992		LTBI	165	162	0.74 [0.67, 0.80]	0.82 [0.75, 0.88]	0.84	N				
41	Darboe 2018	Darboe2018	Darboe11_2018	(GBP2; FCGR1B; GBP5; STAT1; SERPING1; BATF2; GBP1; TRAFD1; SCARF1; TAP1;ETV7	NR	Support vector machine	LTBI	30	30	1.00 [0.88, 1.00]	0.80 [0.61, 0.92]	0.97	N	PBMC	Xpert MTB/RIF	Adults	Indonesia
62	Lesho 2011	Lesho2011	Lesho127_2011	127 transcript signature	NR	Supervised learning algorithms	LTBI+HC	5	18	1.00 [0.48, 1.00]	1.00 [0.81, 1.00]	NR	N	PBMC	Culture	Adults	USA
53	Maertzdorf 2011	Maertzdorf3_2011	Maertzdorf3_2011	CD64; LTF; RAB33A	Maertzdolf-GSE25534	Random Forest	LTBI	32	34	0.88 [0.71, 0.96]	0.91 [0.76, 0.98]	NR	N	Whole Blood	Culture	Adolescents and adults	SA
		Maertzdorf5_2011	Maertzdorf5_2011	FCGR1B; CD64;GBP5 LTF; GZMA	Maertzdolf-GSE25534		LTBI	32	34	0.94 [0.79, 0.99]	0.97 [0.85, 1.00]	NR	N				
39	Satproedprai 2015	Satproedprai2015	Satproedprai7_2015	FCGR1A; FCGR1B variant 1; FCGR1B variant 2; MAFB; APOL1;	NR	Logistic regression	HC	40	56	0.82 [0.67, 0.93]	1.00 [0.94, 1.00]	0.97	N	Whole Blood	Culture + Smear	Adults	Thailand

				STAT1; KAZN													
57	Serrano 2016	Serrano2016	Serrano2_2016	NCF1; ORM	NR	Analysis of variance	LTBI+HC	10	20	0.90 [0.55, 1.00]	0.80 [0.56, 0.94]	NR	N	PBMC	Culture + Smear	Adults	Mexico
61	Wu 2007	Wu2007	Wu3_2007	IL-8; FOXP3; IL-12β,	NR	Logistic regression	LTBI	30	24	0.97 [0.83, 1.00]	0.88 [0.68, 0.97]	0.97	N	PBMC	Culture or CXR	Adults	USA

*AUC; Area under the curve, UK; United Kingdom, USA; United States of America, Sens; Sensitivity, Spec; Specificity, LTBI; Latent TB infection, HHC; Household TB Contact. * Signature discovery method applies to the signature that was discovered in that article.*

Table 2. Characteristics of studies for predicting progression to TB disease included in the systematic review.

Ref.	Study Name	Study Entry ID	Signature Name	Biomarker Description	Dataset	Signature discovery model	Population type	Total enrolled	Progressed to TB	Did not Progress to TB	AUC	Index Sample	TB reference standard	Population	Sample Country
55	Roe 2019	Roe2019b	Roe3_2019	BATF2; GBP5; SCARF1;	Roe-E-MTAB6385	Stability selection	HHC	333	6	327	NR	Whole blood	Culture	Adults	UK
26	Suliman 2018	Suliman2018b	Suliman4_2018	GAS6; SEPT4; CD1C; BLK	Zak-GSE79362	Pair ratio	LTBI	145	41	104	0.69	Whole blood	Culture or 2 Smears	Adults	South Africa
60	Warsinske 2018	Warsinske2018b	Sweeney3_2016	GBP5; DUSP3; KLF2	Zak-GSE79362	Forward search	LTBI	144	43	101	0.86	Whole blood	Culture or 2 Smears	Adolescents	South Africa
25	Zak 2016	Zak2016p	Zak16_2016	Refer to 25 in table 1 above	Suliman-GSE94438	Support vector machine	HHC	374	73	301	0.72	Whole blood	Culture & Smear	Adults	USA

AUC; Area under the curve, UK; United Kingdom, USA; United States of America, LTBI; Latent TB infection, HC; Healthy control, OD; Other diseases

Table 3. Performance of Signatures for predicting progression to TB disease.

Study Name	Signature	Time window before TB (months)	Sensitivity	Specificity	PPV	NPV
Roe2019b	Roe3_2019	12	66.7%	98.8%	52.7%	99.3%
Suliman2018b	Suliman4_2018	24	75.6%	54.8%	3.3%	99.1%
Warsinske2018b	Sweeney3_2016	6	86.0%	84.2%	10.0%	99.7%
Zak2016p	Zak16_2016	12	53.4%	82.7%	5.9%	98.9%

PPV and NPV where calculated at 2% pre-test probability. PPV; Positive predictive value, NPV; Negative predictive value
